# Microbiota and mycobiota in bronchoalveolar lavage fluid of silicosis patients

**DOI:** 10.1186/s12995-023-00377-3

**Published:** 2023-07-10

**Authors:** Linshen Xie, Xiaoyan Zhang, Xiaosi Gao, Linyao Wang, Yiyang Cheng, Shirong Zhang, Ji Yue, Yingru Tang, Yufeng Deng, Baochao Zhang, Xun He, Mingyuan Tang, Hua Yang, Tianli Zheng, Jia You, Xuejiao Song, Jingyuan Xiong, Haojiang Zuo, Xiaofang Pei

**Affiliations:** 1 West China School of Public Health and West China Fourth Hospital, Sichuan University, Chengdu, 610041 China; 2 Food Safety Monitoring and Risk Assessment Key Laboratory of Sichuan Province, Chengdu, 610041 China

**Keywords:** Silicosis, BALF, Microbiota, Mycobiota, Fatigue, *Vibrio*

## Abstract

**Background:**

The contribution of bronchoalveolar lavage fluid (BALF) microbiota and mycobiota to silicosis has recently been noticed. However, many confounding factors can influence the accuracy of BALF microbiota and mycobiota studies, resulting in inconsistencies in the published results. In this cross-sectional study, we systematically investigated the effects of “sampling in different rounds of BALF” on its microbiota and mycobiota. We further explored the relationship between silicosis fatigue and the microbiota and mycobiota.

**Methods:**

After obtaining approval from the ethics board, we collected 100 BALF samples from 10 patients with silicosis. Demographic data, clinical information, and blood test results were also collected from each patient. The characteristics of the microbiota and mycobiota were defined using next-generation sequencing. However, no non-silicosis referent group was examined, which was a major limitation of this study.

**Results:**

Our analysis indicated that subsampling from different rounds of BALF did not affect the alpha- and beta-diversities of microbial and fungal communities when the centrifuged BALF sediment was sufficient for DNA extraction. In contrast, fatigue status significantly influenced the beta-diversity of microbes and fungi (Principal Coordinates Analysis, *P* = 0.001; *P* = 0.002). The abundance of *Vibrio* alone could distinguish silicosis patients with fatigue from those without fatigue (area under the curve = 0.938, 95% confidence interval [CI] 0.870–1.000). Significant correlations were found between *Vibrio* and haemoglobin levels (*P* < 0.001, ρ = -0.64).

**Conclusions:**

Sampling in different rounds of BALF showed minimal effect on BALF microbial and fungal diversities; the first round of BALF collection was recommended for microbial and fungal analyses for convenience. In addition, *Vibrio* may be a potential biomarker for silicosis fatigue screening.

**Supplementary Information:**

The online version contains supplementary material available at 10.1186/s12995-023-00377-3.

## Background

Silicosis, a common type of pneumoconiosis, is a work-related fibrotic lung disease usually caused by occupational exposure to respirable crystalline silica and hazardous dust [1]. Crystalline silica is a common component of the earth’s crust and sand. Occupational exposure occurs mainly in the construction and stone-working industries [2, 3]. When respirable crystalline silica is inhaled, it can trigger inflammatory processes and the production of reactive oxygen species, which can lead to fibrogenesis and carcinogenesis [3].

It is estimated that 5.5 million workers in Europe [4], 2.3 million in the United States [3], and 11.5 million in India [5] are regularly exposed to respirable crystalline silica. Although prevention and control measures have substantially contributed to tackling this problem, China is still thought to have the largest number of silicosis cases, with 11,809 new pneumoconioses and approximately 6,000 new silicosis reported annually [5]. Sichuan has a high incidence of silicosis and pneumoconiosis among the provinces in China [6]. It is one of the most important provinces in Southwest China, with a population of 83 million, a total area of 486,000 square kilometres, and a GDP of approximately 700 USD (4859.88 RMB) billion, mainly from the secondary sector [7, 8]. While silicosis is progressive, irreversible, and incurable, treatment options for severe silicosis remain limited [9], causing a considerable medical burden in Sichuan.

In recent decades, lung-gut axis studies have found that microbiota may be related to occupational health [10–12]. A 16S rRNA gene sequencing study examined gut microbial composition from 18 patients with silicosis and 21 healthy subjects. The results revealed significant alterations in bacterial composition. Patients with silicosis had lower bacterial diversity in their intestinal microbiota than that of the subjects. This reduction in diversity was accompanied by an increase in *Proteobacteria* abundance [10]. Qi et al. found that silica-exposed rats with severe pulmonary fibrosis were accompanied by gut microbiota dysbiosis (aberrant populations) and significantly decreased *Bifidobacterium* [12]. Further research indicated that lung injury induced by silica exposure may affect gut microbiota disturbance through lung inflammation [11, 12]. Lung immune homeostasis is balanced by microbiota (bacterial microbiome) and mycobiota (fungal microbiome) [13]. Dysbiosis of the lung microbiome has been observed in many pulmonary diseases that are not traditionally considered microbial in origin [14]. It may also be crucial in the development of silicosis [15]. However, whether aberrant populations of lung microbiota, in combination with mycobiota, contribute to silicosis is unclear. Moreover, little is known about the stability of the lower airway microbiome or the representative sampling method for the bronchoalveolar lavage fluid (BALF) microbiota and mycobiota analyses of patients with silicosis.

Lung lavage is suggested for the symptom-relief treatment for silicosis [16, 17]. In patients with silicosis, lavage of both lungs generally takes more than 2 h, requiring up to 20 L of normal saline. Usually, the sample volume required for microbiological and cytological examinations is less than 1 L. Therefore, collecting a few representative samples for measurement from the 20 L lung lavage fluid is important, to ensure the reproducibility and comparability of the results, is particularly important. Robinson et al. [18] used the sampling scheme of lung lavage fluid for cell counting and found that for the same individual, the number of cells collected at different time points of lung lavage varied considerably; cells collected from the first 50 mL can provide more accurate cell counts. This study suggested that the standardising the lung lavage fluid sampling protocol can help improve the accuracy and repeatability of the analysis results.

Currently, there is still a lack of a standard sampling plan to ensure the quality of the analysis of BALF microbiota and mycobiota, which also makes cross-comparison of the results of different studies very difficult. In contrast, there are many types of bacteria in lung lavage fluid, the niches and adhesions of different types of bacteria are different, and their elution time may also be different. For example, *Prevotella* spp. are part of the normal human lung microbiota. They can adhere to lung epithelial cells and have been reported to modulate immune responses [19]. Moreover, *Lactobacillus* spp. [20] and *Bifidobacterium* spp. [21], which are commonly associated with the gut microbiome, have also been found in the respiratory tract. These bacteria have been shown to exert immunomodulatory effects by adhering to lung epithelial cells. In healthy individuals, the fungi *Malassezia* spp. [22] and *Candida* spp. [23] are also considered to be a normal commensal of the skin, respiratory tract, and other body sites. *Candida* spp. adheres to lung epithelial cells using adhesins such as agglutinin-like sequence (ALS) proteins [24]. In addition, the exopolysaccharide galactosaminogalactan of the fungal pathogen *Aspergillus* spp. is involved in adhesion to lung epithelial cells. Researchers also found that this exopolysaccharide contributes to virulence by enhancing the fungus's resistance to neutrophil extracellular traps, which are part of the host immune response [25]. Thus, the adhesion properties of bacteria and fungi in the lung might vary greatly. Additionally, these properties are not well-characterised.

Therefore, in the context of the vigorous development of precision medicine, for the analyses of the microbiota and mycobiota in the lung lavage fluid, a standardized sampling is imperative to assure quality control. Therefore, we used 16S ribosomal ribonucleic acid (rRNA) gene sequencing [26] and internal transcribed spacer 1 (ITS1) sequencing [27] to systematically investigate the effects of “sampling in different rounds of BALF” on its microbiota and mycobiota. We further explored the relationship among BALF microbiota, mycobiota, and silicosis fatigue.

## Methods

### Study participants and study design

This study was conducted at West China Fourth Hospital, the only national occupational disease hospital in China, where the operation of large-volume BALF treatments (20L) is unsurpassed in China in terms of quantity and quality. The cross-sectional study population included men diagnosed with silicosis, according to the Diagnosis of Occupational Pneumoconiosis (GBZ 70–2015) clinical guideline, mainly from the core area of Sichuan [7, 8]. The inclusion criteria for participants in this study were patients who were: 1) adult and male with stage I chronic simple silicosis [28], 2) scheduled for lung lavage treatment, and 3) without medical history of tuberculosis. To avoid potential bias, participants were excluded if 1) antibiotics were used within 4 weeks or 2) the library failed sequencing due to insufficient DNA quantity, PCR amplification failure, etc.

As a pilot study, homogeneous patients with the same stage of chronic silicosis were chosen. A total of 10 consecutive patients with silicosis, including 100 samples and approximately 200 microbiota and mycobiota results were obtained from 2019 to 2020. All samples were sequenced in the same batch to exclude batch-to-batch variation. According to previous studies, each subgroup contained 3–9 BALF samples [29, 30], indicating that our sample size would be acceptable in this pilot study.

Before performing BALF, demographics; blood tests; radiological examination; lung function; Barthel index [31]; symptoms such as cough, expectoration, chest pain, fever, hemoptysis and fatigue levels of the patients were collected. According to the Fatigue Severity Scale, an average score of ≥ 4.0 indicates a clinically significant level of fatigue [32]. Notably, half of the patients experienced fatigue, while the other half did not. The Institutional Review Board of West China School of Public Health/West China Fourth Hospital, Sichuan University, approved this study (IRB Number: HXSY-EC-2020073). All subjects signed an informed consent form before lung lavage. The Institutional Review Board waived the requirement for written informed consent for this study. The information has been sufficiently anonymised. Neither the patient nor anyone else could identify the patient.

### Procedures and sample collection

**Fig. 1:**
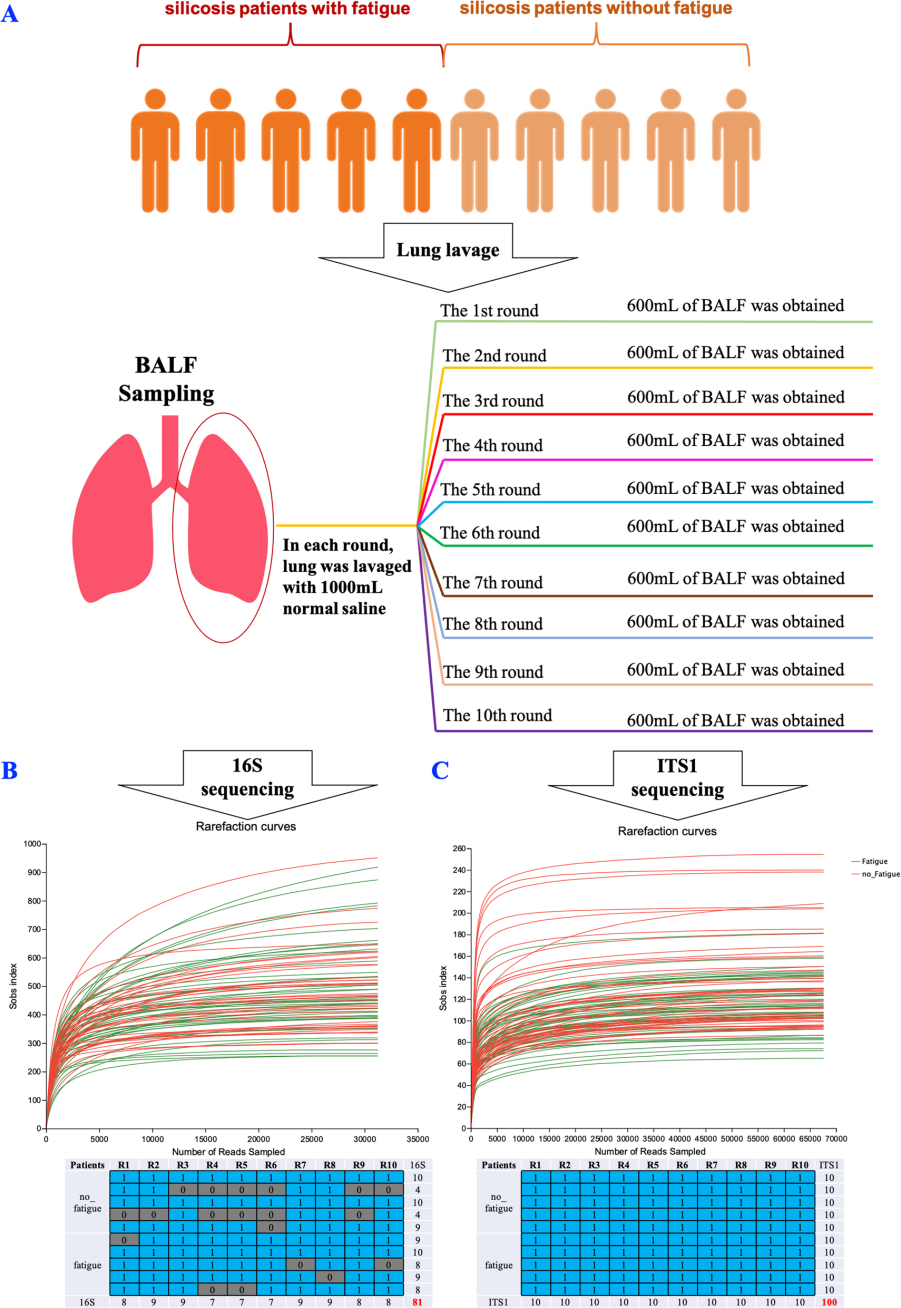
Schematic diagram of this study. **A** sampling in different rounds of BALF. **B** BALF 16 s rRNA gene sequencing (*n* = 81). C: BALF ITS1 gene sequencing (*n* = 100)

After general anaesthesia, lung lavage was performed by specialists. Each lung was flushed ten times with 1 L sterile normal saline at 37 ℃. In each round, 600 mL lavage fluid was collected from the left lung (Fig. 1A). The samples were placed on ice and transported to the laboratory within an hour. Each sample was centrifuged in 50 mL centrifuge tubes under the condition at 13,000 g and 4℃ for 2 min. The sediment was then stored at − 80 ℃.

### DNA extraction

Total genomic DNA samples were extracted using the Zymo Bacterial/Fungal DNA Mini-Prep Kit (Zymo Research, Irvine, CA, USA), following the manufacturer’s instructions, and stored at -80 °C prior to further analysis. The quantity and quality of the extracted DNA were measured using a NanoDrop ND-1000 spectrophotometer (Thermo Fisher Scientific, Waltham, MA, USA) and agarose gel electrophoresis, respectively.

### Library construction, sequencing, and analyses of microbiota and mycobiota

The 16S rRNA gene was amplified using barcoded 338 F:5′-ACTCCTACGGGAGGCAGCA-3′ and 806R:5’-GGACTACHVGGGTWTCTAAT-3’ [33]. The ITS1 gene was amplified using barcoded ITS1 5F:5′-GGAAGTAAAAGTCGTAACAAGG-3′ and ITS1 2R:5′-GCTGCGTTCTTCATCGATGC-3′ [27, 34]. DNA libraries were constructed according to protocol described in a previous study [35]. Amplicons were pooled and sequenced on an Illumina NovaSeq PE250 (Illumina, Inc., San Diego, CA, USA) platform. The sequencing data were processed with integrated pipelines for bioinformatics analysis [36].

The alpha diversity was calculated to evaluate community diversity in the sample (or habitat). Chao, Ace, and Sobs indices reflect the species richness of the community. According to the sequencing results, the Sobs index refers to the actual observed species number at a certain sampling level, while Chao and Ace use the chao1 and Ace algorithms, respectively, to estimate the total number of species at a certain sampling level. The larger their values, the more types of species exist in the habitat. The Shannon and Simpson indices can mirror the species diversity of the community, and are affected by both species evenness and species richness [37]. The larger the Shannon value, the higher the community diversity. The larger the Simpson index value, the lower the community diversity [36, 38].

Beta diversity analysis focuses on the differences between the groups (between the samples). Through group comparative analysis of species diversity among different habitats or microbial communities, we can explore the similarity and differences in community composition among the different grouping samples. The main analysis methods include principal component analysis (PCA) and principal coordinate analysis (PCoA). This study used PCA and PCoA to explain the differences in diversity between the groups. PCA reduces the dimensionality of complex variables through unsupervised multivariate statistical methods; moreover, it simplifies and obtains several unrelated new comprehensive variables (i.e., principal components). This can reflect the overall differences among the samples of each group and the variation among the samples within a group. In the PCA, the greater the difference among the microbiota of each sample, the farther away they are from each other and vice versa. PCoA is similar to PCA. The main difference is that PCA uses the abundance table of species [including amplicon sequence variants (ASV)] and is directly related to the Euclidean distance. While, the PCoA is drawn based on the selected distance matrix. Both of them use dimensionality reduction to find potential principal components that affect the differences in sample community composition [38, 39].

Linear discriminant analysis (LDA) with effect size measurement (LefSe) was used to test the discriminatorily abundant taxonomic characteristics between the different groups. Microbial taxa with LDA scores > 2 and a *P*-value < 0.05 were recognised as significantly different. Taxa at different levels were also evaluated by the Wilcoxon rank-sum test. A random forest model was constructed to explore the potential of the lung microbiota/mycobiota to distinguish silicosis patients with fatigue from those without fatigue. The model’s performance was assessed by applying the receiving operational curve (ROC) analysis. Phylogenetic Investigation of Communities by Reconstruction of Unobserved States 2 (PICRUSt2) was also applied to infer the association between lung microbial/mycobial functions and silicosis [36].

### Statistical analysis

Statistical analyses were performed using the R software (version 4.1.1, R Core Team, Vienna, Austria) and SPSS (v23.0, SAGE IBM, Armonk, NY, USA). Chi-square analysis and Fisher’s exact tests were used for categorical variables, whereas the t-test and Wilcoxon rank-sum test and Kruskal–Wallis H test were used for continuous variables. All the tests were two-sided, and *P*-values < 0.05 were considered statistically significant. For microbiota and mycobiota analyses, the *P*-values were adjusted for the false discovery rate (FDR).

## Results

### Impact of “Sampling in different rounds of BALF” on microbiota and mycobiota profiles

A total of 100 BALF samples were collected from 10 consecutive patients with silicosis from the West China Fourth Hospital. The basic patient information is presented in Supplementary Table 1. We randomly chose 30,981 and 67,698 qualified reads from each sample for the microbiota (Fig. 1B) and mycobiota (Fig. 1C) analyses, respectively. Finally, 2,509,461 (30,981 reads/sample × 81) and 6,769,800 (67,698 reads/sample × 100) ASV were obtained for the microbiota and mycobiota analyses, respectively.

The BALF samples from rounds 1 to 10 were categorised in three different ways—(1) into two groups, including the first five (first half, 50%) and the last five (second half, 50%) rounds; (2) into three groups, including the first third (30%), the middle third (40%), and the last third (30%); and (3) into 10 groups.

#### Alpha-diversity and community composition analyses

Figures S1-S4 present the differences in microbial and fungal alpha-diversity and community composition of BALF obtained from different rounds of lavage. We found that sampling from the BALF obtained from different rounds of lavage did not significantly influence the variability in alpha-diversity, including the Chao, ACE, Sob, Shannon, and Simpson indices (Figures S1A-O and S2A-O). Meanwhile, no noticeable differences were observed in the compositions of microbial and fungal communities in the BALF obtained from different rounds of lavage (Figures S3A-L and S4A-L).

#### Beta-diversity analysis

Figures S5 and S6 show the differences in microbial and fungal beta-diversity in the BALF obtained from different rounds of lavage. Sampling from the BALF obtained from different rounds of lavage had no apparent effect on the variability in beta-diversity, as revealed by the PCA (Figures S5A, C, E and S6A, C, E) and PCoA (Figures S5B, D, F and S6B, D, F).

#### LefSe analysis

The LefSe was used to select the most significant differences in taxa among different groups. Figures S7A-I and S8A-I depict the differences in microbial and fungal taxa present in the BALF obtained from different rounds of lavage, respectively. Based on LDA selection, only a small part of the specific microbiota (p_*Verrucomicrobiota*, p_*Deinococcota*, g_*Gemella*, and g_*Bifidobacterium*) and mycobiota (g_*Agaricus*, g_*Fusidium*, g_*Knufia*, and g_*Cylindrobasidium*) changed significantly, depending on the sampling round.

#### Functional prediction

Microbial and fungal function profiles of silicosis patients with and without fatigue were analysed using Phylogenetic Investigation of Communities by Reconstruction of Unobserved States 2 (PICRUSt2) (The Huttenhower Lab, Boston, MA, USA), the Wilcoxon rank-sum test and FDR adjustment. No significant difference was observed in the BALF obtained from different rounds of lavage.

### Impact of fatigue on BALF microbiota and mycobiota profiles

**Fig. 2:**
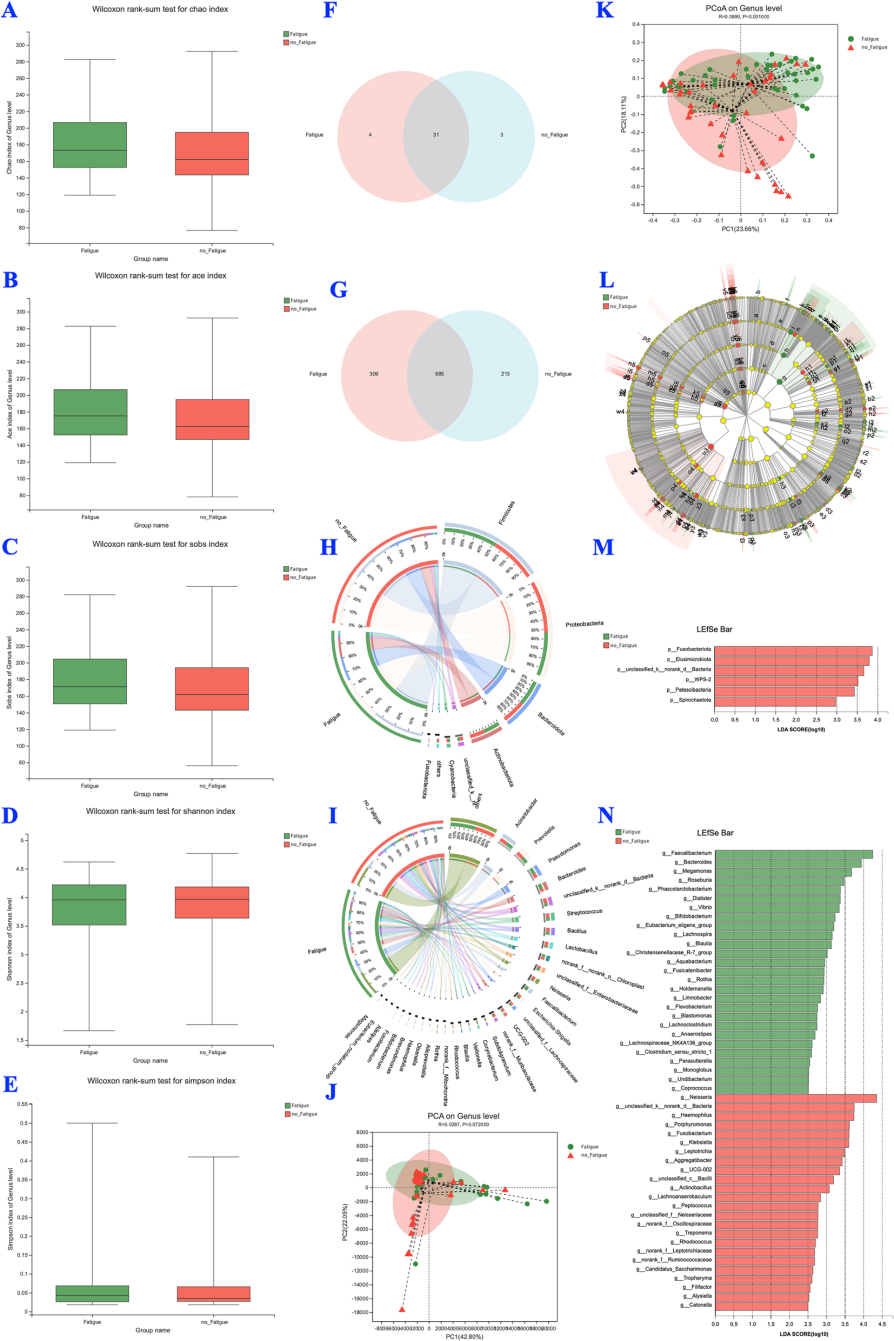
BALF microbiota profiles of silicosis patients with and without fatigue (Fatigue vs. no Fatigue, *n* = 81). **A**-**E** α diversity analyses; **F** Venn analysis at the phylum level; **G** Venn analysis at the genus level; **H** Circos analysis at the phylum level; **I** Circos analysis at the genus level; **J** PCA analysis at the genus level; **K** PCoA analysis at the genus level; **L** Cladograms of LefSe from phylum to genus; **M** LefSe analysis at the phylum level; **N** LefSe analysis at the genus level

**Fig. 3:**
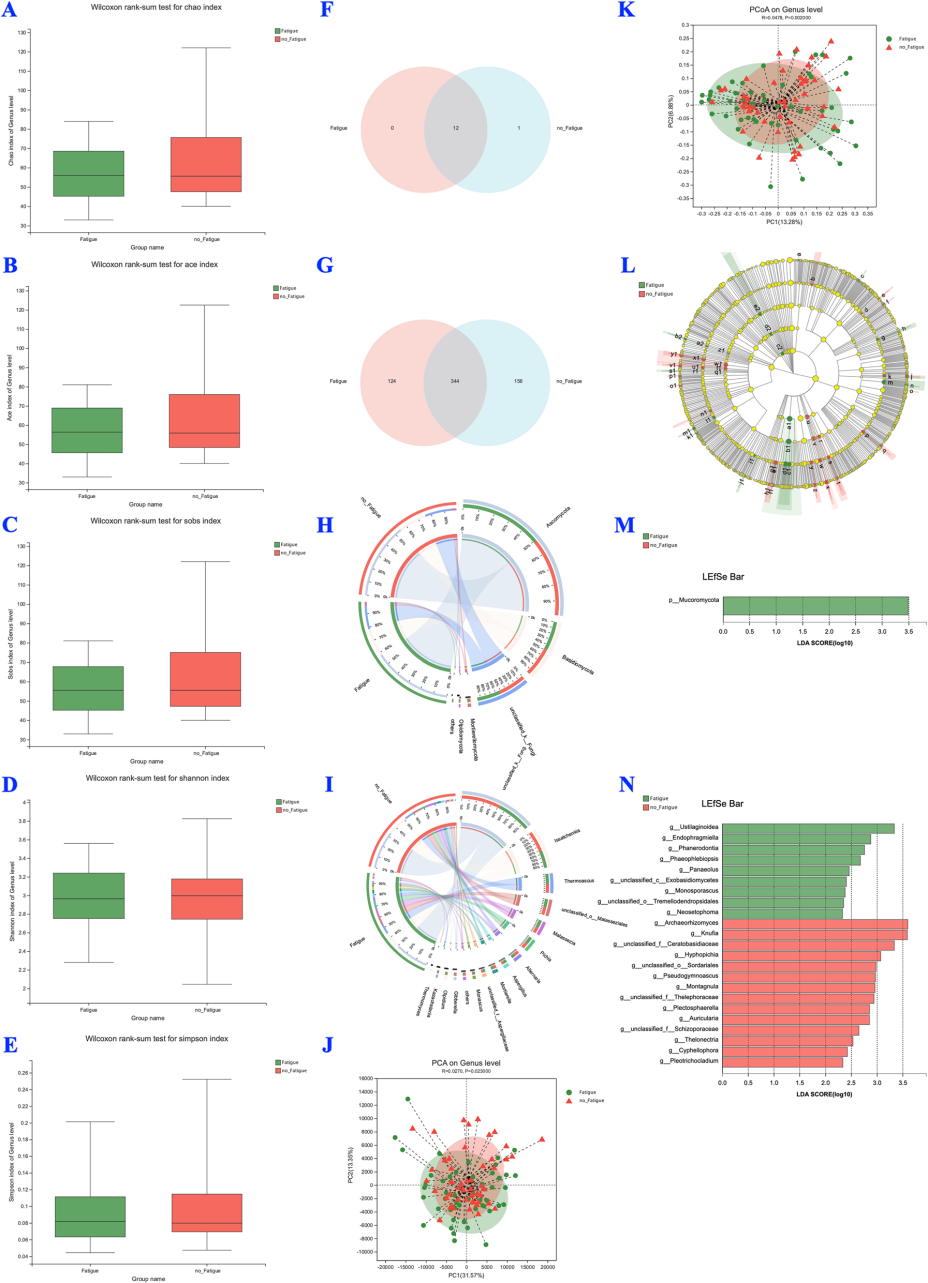
BALF mycobiota profiles of silicosis patients with and without fatigue (Fatigue vs. no Fatigue, *n* = 100). **A**-**E** α diversity analyses; **F** Venn analysis at the phylum level; **G** Venn analysis at the genus level; **H** Circos analysis at the phylum level; **I** Circos analysis at the genus level; **J** PCA analysis at the genus level; **K** PCoA analysis at the genus level; **L** Cladograms of LefSe from phylum to genus; **M** LefSe analysis at the phylum level; **N**: LefSe analysis at the genus level

**Fig. 4:**
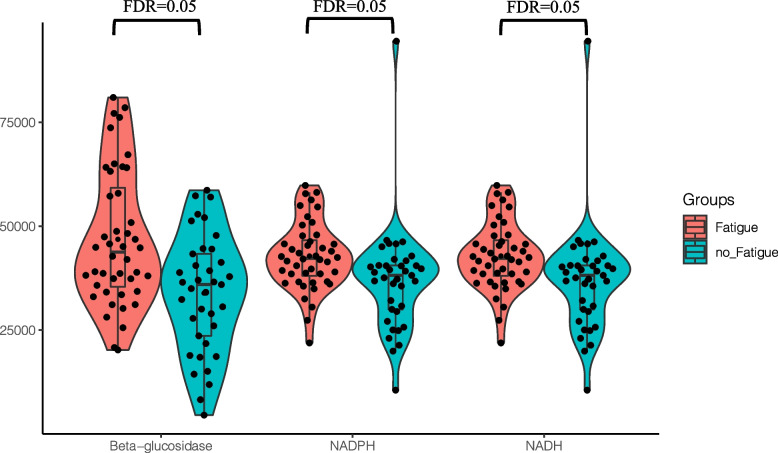
PICRUSt2 prediction analysis of silicosis patients with and without fatigue (Fatigue vs. no Fatigue, *n* = 81)

**Fig. 5:**
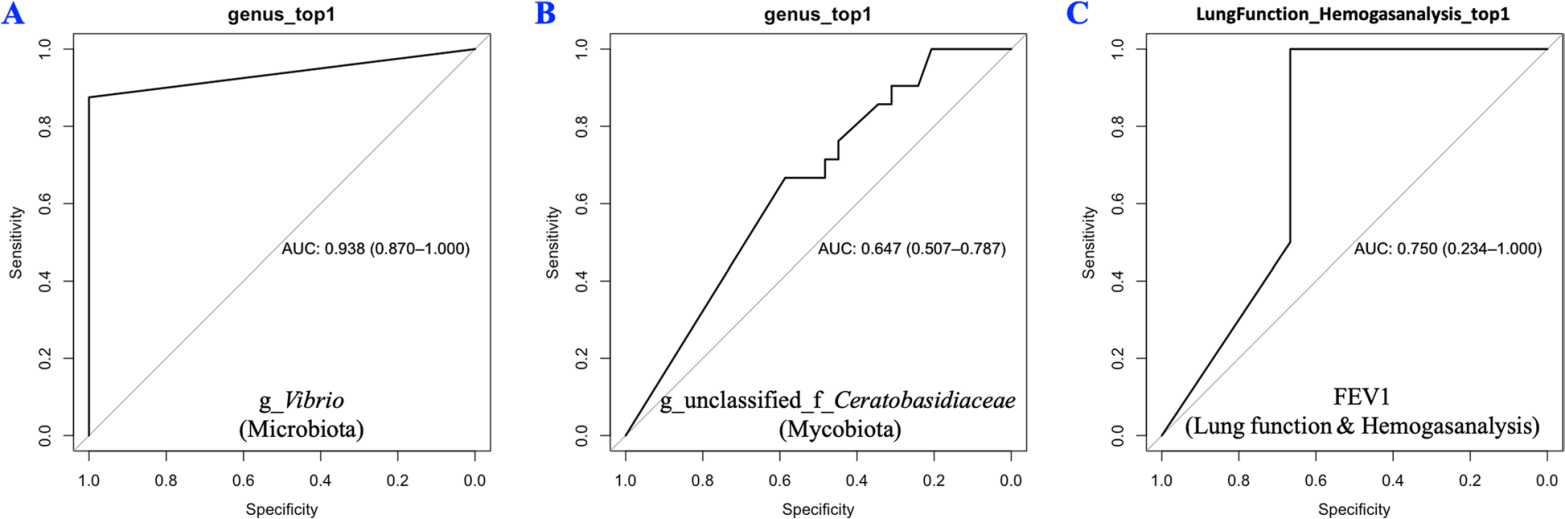
Classification power of potential microbial (**A**), fungal (**B**) and lung fuction & hemagasanalysis (**C**) markers associated with fatigue by ROC analyses. Eight lung function and hemagasanalysis parameters, namely, (1) FVC%Pred(%); (2) FEV1%Pred(%); (3) FEV1%FVC Pred(%); (4) DLCO%Pred(%); (5) pH; (6) sO_2_ (%); (7) pO_2_ (mmHg); (8) pCO_2_ (mmHg) were included for ROC analysis. Similar to the situation of microbiota and mycobiota, due to the change trends of each parameter in the "fatigue group" and "no fatigue group" being different, only one parameter with the highest resolution (top1) is selected from eight parameters. The result demonstrated that FEV1 has the highest resolution. ROC, receiving operational curve; AUC, area under curve; FVC, forced vital capacity; Pred, predicted; FEV1, forced expiratory volume in 1 s; DLCO, diffusing capacity of the lung for carbon monoxide; pH, acidity; sO_2_, Oxygen saturation; pO_2_, partial pressure of oxygen; pCO_2_, partial pressure of carbon dioxide

Figures 2, 3, 4 and 5 demonstrate the differences in the microbial and fungal characteristics of silicosis patients with and without fatigue.

#### Alpha-diversity and community composition analyses

No significant difference was observed in the alpha-diversity of the microbiota of patients with and without fatigue (Fig. 2A-E). The two groups shared 31 phyla and 685 genera (Fig. 2F and G). They also had the same levels of unique phyla (4 vs. 3, Fig. 2F) and unique genera (309 vs. 213, Fig. 2G). Circos analysis indicated no noticeable differences in the microbial community composition at the phylum (Fig. 2H) and genus levels (Fig. 2I) between patients with and without fatigue.

No significant difference was observed in the alpha-diversity of the mycobiota of patients with and without fatigue (Fig. 3A-E). The two groups shared 12 phyla and 344 genera (Fig. 3F and G), while having the same levels of unique phyla (0 vs. 1, Fig. 3F) and unique genera (124 vs. 156, Fig. 3G). Circos analysis indicated no apparent differences in the fungal community composition at the phylum (Fig. 3H) and genus levels (Fig. 3I) between patients with and without fatigue.

#### Beta-diversity analysis

The results of the PCA (*P* = 0.07, Fig. 2J) and PCoA (*P* = 0.001, Fig. 2K) indicated that the microbial communities in silicosis patients with and without fatigue were partly separated. The beta-diversity analysis [PCA (*P* = 0.02, Fig. 3J) and PCoA (*P* = 0.001, Fig. 3K)] of mycobiota showed similar results.

#### LefSe analysis

Among the microbiota, *Vibrio*, *Faecalibacterium*, and *Dialister* (Fig. 2N) were significantly enriched, while *Fusobaceriota* (Fig. 2M) and *Rhodococcus* (Fig. 2N) abundance was remarkably reduced in the fatigue group compared with that in the silicosis patients without fatigue. Among the mycobiota, the abundance of p_*Mucoromycota* (Fig. 3M), g_*Ustilaginoidea*, g_*Archaeorhizomyces*, and g_unclassified_f_*Ceratobasidiaceae* (Fig. 3N) significantly differed between silicosis patients with and without fatigue.

#### Functional prediction

No significant difference between silicosis patients with and without fatigue was observed in the Kyoto Encyclopedia of Genes and Genomes (KEGG) categories (Table S2). However, enzyme analyses revealed that the fatigue group had higher beta-glucosidase (46,842.51 ± 16,170.78 vs. 33,881.42 ± 14,172.59), glutamate synthase (nicotinamide adenine dinucleotide phosphate, 42,830.8 ± 8,178.7 vs. 36,980.5 ± 12,895.0), and glutamate synthase levels (nicotinamide adenine dinucleotide, 42,830.8 ± 8,178.7 vs. 36980.5 ± 12895.0) than the silicosis patients without fatigue (Fig. 4, Table S3). Among the mycobiota, no significant difference in enzyme analysis was observed between silicosis patients with and without fatigue (Table S4).

#### ROC analyses

To explore the role of BALF microbiota in predicting fatigue, random forest construction and ROC analysis were performed. The abundance of the genus *Vibrio* alone could distinguish silicosis patients with fatigue from those without fatigue (area under the curve [AUC] = 0.938, 95% confidence interval [CI] 0.870–1.000; Fig. 5A). However, no apparent classification power was observed in the mycobiota analysis (Fig. 5B). The parameters of lung function and hemagasanalysis could also distinguish between silicosis patients with from those without fatigue (AUC = 0.750, 95% CI 0.234–1.000; Fig. 5C).

### Spearman correlation analyses between microbiota and mycobiota

Among all participants, at the genus level (Supplementary Figure S13), significant correlations were observed between *Vibrio* and g_unclassified_f_*Ceratobasidiaceae* (*P* = 0.008, ρ =  − 0.29), g_unclassified_p_*Rozellomycota* (*P* < 0.001, ρ = 0.46), g_*Cladosporium *(*P* < 0.001, ρ = 0.41),g_*Talaromyces* (*P* = 0.004, ρ = 0.32), g_*Olpidium* (*P* = 0.026, ρ =  − 0.25), and g_*Malassezia* (*P* = 0.049, ρ = 0.22). Significant correlations were also observed among the other four taxon levels, i.e., at the phylum (Figure S9), class (Figure S10), order (Figure S11), and family (Figure S12) levels.

### Spearman correlation analyses between microbiota, mycobiota and blood test results

Spearman correlation analyses revealed significant correlations between *Vibrio* abundance and haemoglobin levels (*P* < 0.001, ρ = -0.64, Figure S14) and between *g__unclassified_p__Rozellomycota* abundance and haemoglobin levels (*P* < 0.001, ρ = -0.43, Figure S15).

## Discussion

### Sampling in different rounds of BALF

There is growing evidence of an interaction between the BALF microbiome and silicosis [40]. Although microbial studies have increased rapidly, there is no consensus on the quality control of BALF microbiota and mycobiota collection. Iwasaki et al. reported that BALF was performed by wedging a bronchofibrescope into the bronchi with the largest number of lesions and infusing 50 mL of physiological saline thrice successively. The collected BALF samples (20 mL) were stored at − 80 °C for sequencing [29]. However, Seixas et al. reported that BALF samples had a minimum volume of 15 mL (0.9% saline solution) and were stored at − 80 °C until needed [41]. These articles did not mention centrifugation of the BALF samples. Since bacterial densities in BALF are relatively low, Schneeberger et al. recommended pre-screening of sample bacterial densities [42]. Since different collection methods may introduce experimental variation, especially for large-scale investigations with numerous participants, our major goal was to provide a reference for convenient and reliable BALF sampling to guarantee microbial and fungal study accuracy.

In this study, each sample was first centrifuged in 50 mL centrifuge tubes under the condition of 13,000 g for 2 min at 4 ℃, guaranteeing a sample amount. Then, the sediment was stored at -80 ℃ for further DNA extraction, library construction, and sequencing. Based on 16 s sequencing and ITS1 sequencing analysis, we explored the overall characteristics of the lung lavage fluid of silicosis patients and the changes in the bacterial flora structure during lung lavage (Fig. 1). Our study showed that alpha diversity (Figures S2–S3) and overall microbial and fungal structures (Figures S5–S6) did not significantly differ on subsampling the lung lavage fluids obtained across the different rounds. Less than 2.2% (23/1027) of the identified microbiota at the genus level and 1.6% (10/621) of the mycobiota varied significantly across different rounds of BALF (Figures S7–S8). Sampling in different rounds of BALF had a minimal effect on the diversity of BALF microbiota and mycobiota. Thus, considering the convenience of sampling, the first round of BALF collection is recommended for microbial and fungal analyses. Our study is the first, to our knowledge, to provide a reference for standardising the sampling of the lung lavage fluid of patients with silicosis to avoid experimental bias.

Adhesion variations of the specific BALF microbiota and mycobiota might be reflected by the results of the LEfSe analysis (Figures S7 and S8). At the phylum level, we found that the abundance of two phyla, *Verrucomicrobiota* and *Deinococcota*, was significantly higher in the last five rounds than in the first five rounds (Figure S7B). At the genus level, we found that *Gemella*, etc. were significantly higher in the first five rounds than in the last five rounds, while *Bifidobacterium*, etc. were significantly higher in the last five rounds than in the first five rounds. *Gemella* is found at the mucosal surface of the aerodigestive tract. Druzhinin et al. observed an increase in the representation of the genera *Gemella*, *Streptococcus*, and *Bacillus* in the sputum of miners with coal workers’ pneumoconiosis compared to that of control subjects [43]. Moreover, *Gemella* might play a direct role in and/or may be a biomarker for the exacerbations of chronic lung disease [44]. Among patients with idiopathic pulmonary fibrosis (IPF), Dickson et al. compared BALF microbiota of IPF patients with and without honeycombing, and found a potential association between *Gemella* spp. and honeycombing [45]. It is speculated that honeycombing alters community composition, contributes to microbiota growth, such as *Gemella* spp., and causes injury from mucin overexpression and defective mucociliary clearance [45]. For *Bifidobacterium*, Chen et al. compared the microbial communities in lung cancer tissues between patients with and without lymph node metastasis. A higher abundance of *Bifidobacterium* was observed in the lymph node metastasis group [46]. However, it is currently difficult to find studies examining *Bifidobacterium* or mycobiota in lung silicosis. In this study, the microbiota and mycobiota composition of BALF may be help in clarifying the role of microbiota in lung fibrosis.

### Fatigue, *Vibrio* and haemoglobin

Fatigue is a major symptom of silicosis [47]. It significantly affects the work efficiency and quality of life of patients and has become a severe health hazard in occupational diseases worldwide [48]. The microbiota produce various mediators that can travel in distant ways and affect the health of the host negatively or positively. Similar to other microbial studies [49], we found an emerging role of BALF microbiota, especially *Vibrio*, in the fatigue symptoms of patients with silicosis. In our study, the fatigue status was significantly correlated with the overall variability of microbial and fungal structures (*P* = 0.001, Fig. 2K; *P* = 0.002, Fig. 3K). The abundance of the genus *Vibrio* alone was able to distinguish silicosis patients with fatigue from those without it (AUC = 0.938, 95% CI 0.870–1.000, Fig. 5A). Significant correlations were also found between *Vibrio* and haemoglobin (*P* < 0.001, ρ = -0.64, Figure S14).

Studies have shown that a decrease in beneficial bacteria and an increase in pathogenic bacteria coexist when fatigue is present in both rodents and humans [50]. *Vibrio* thrives in many moist environments, including the respiratory system. However, it is not considered a major member of the healthy human lung microbiota [51]. It can be isolated from the lungs of animals with chronic obstructive pulmonary disease [52] and patients with non-small cell lung cancer [51]. Dong et al. also reported that the relative abundance of *Vibrio* was increased in immunosuppressed rats, compared with that in the control group [53]. In addition, opportunistic and pathogenic *Vibrio* infections can cause many diseases [54].

Our findings support the results of Cella et al. in that a significantly positive relationship between haemoglobin rise and fatigue reduction was observed [55]. Moreover, Krishnan et al. reported that increased haemoglobin levels were associated with clinically significant improvements in fatigue [56]. Tardy et al. also suggested that when the levels of haemoglobin (the oxygen carrier) are decreased, oxygen delivery is impaired, which might result in fatigue and tiredness [57].

We did not find direct mechanisms responsible for the correlations among *Vibrio*, haemoglobin, and fatigue. A possible mechanism is that iron is an indispensable element in the growth and metabolism of some *Vibrio* species, and iron in the host cells is mainly found in the haemoglobin of red blood cells [58]. Some *Vibrio* species can obtain iron from haemoglobin-related complexes [59]. In addition, among the harmful effects of fatigue, oxidative stress and inflammation have also been observed [60]. Our findings enable deeper insight into the therapeutic potential of alternating the BALF microbiota, especially *Vibrio*, to alleviate fatigue in patients with silicosis [61–63]. However, the relationship between BALF *Vibrio*, fatigue, and haemoglobin remains to be elucidated.

Because fatigue in silicosis may be due to the degree of lung function, gas exchange impairment, or the extent of radiological alterations. Accordingly, ROC analysis was performed using lung function and hemogasanalysis parameters to distinguish silicosis patients with fatigue from those without fatigue. Its results were compared with those attained using the abundance of BALF microbiota and mycobiota. This comparison has clinical relevance since it provides evidence of whether an invasive, complex, and costly (approximately 15 USD per sample) method, such as BALF microbiota analysis, is actually necessary to predict fatigue in silicosis patients. We found that the genus *Vibrio* alone could distinguish silicosis patients with fatigue from those without fatigue (AUC = 0.938, Fig. 5A), which was comparatively more effective than that of the functional and hemogasanalysis parameters (AUC = 0.750, Fig. 5C). In addition, selective modulation of lung microbiota might offer a novel therapeutic approach to relieve silicosis-induced fatigue and improve patients’ quality of life [64, 65]. The role and significance of the lung microbiome in diagnosing, predicting, and treating silicosis need to be explored. Furthermore, potential behavioural and/or microbiome-related preventive measures could be further derived to help maintain employees' health [66].

We did not find a significant difference in the alpha diversity of lung microbiome between silicosis patients with and without fatigue. This alpha diversity is associated with lung immune homeostasis and lung diseases [67]. It was found to be significantly higher in the control than in tumour lung samples [67–69]. In contrast, no significant change in bacterial diversity after smoking cessation was observed, suggesting that smoking may not play a major role in altering the composition of lung commensal bacteria [67, 70]. In general, the research on the lung microbial community is still in its early stages; moreover, it is not yet possible to simply classify the level of the diversity index as a positive or negative factor. To draw accurate conclusions, further in-depth research is needed on the relationship between lung microbial diversity and silicosis.

### Strengths and limitations

Our study has several strengths. First, this study was conducted at West China Fourth Hospital, China's only national occupational disease hospital, where the operation of large-volume BALF treatments (20L) is unsurpassed in China in terms of quantity and quality. Second, the effects of sampling in different rounds of BALF were analysed. This study might provide an essential reference for microbiome sampling from BALF. Third, to the best of our knowledge, no previous study has investigated both microbial and fungal data in patients with silicosis. Meanwhile, our rarefaction depth exceeds the general requirement by 3 times, elaborated as follows. In microbiome analysis, rarefaction is used to standardise the sequencing depth of different samples, so that they can be compared and analysed [71]. Rarefaction curves are typically calculated using the sample with the lowest number of ASVs as the standard. This approach ensures that all samples are rarefied to the same number of ASVs, avoiding bias due to the differences in sequencing depth and minimising the loss of information. Meanwhile, the default parameter of rarefaction depth is usually set as 10,000 reads per sample [72]. In our study, the smallest read numbers were 30,981 and 67,698 for the microbial and fungal samples, respectively. Therefore, we randomly chose 30,981 (Fig. 1B) and 67,698 (Fig. 1C) qualified reads from each sample for the microbiota and mycobiota analyses, respectively. Thus, the rarefaction depth of this study exceeded the required value by 3 times, which might provide a sensitive analysis platform to assess the relative abundance of different microbiomes [73]. In addition, the relationship between *Vibrio* and fatigue (Fig. 5A) was partly supported by the relationship between *Vibrio* and haemoglobin levels (*P* < 0.001, ρ = -0.64, Figure S14) [55], which indicated that our results were reasonable.

Nevertheless, our study had some limitations. First, the lack of a non-silicosis referent group is a major limitation of this study. Because our application for lung lavage in healthy people was not ethically approved in this pilot study. Besides, Silicosis is an interstitial lung disease caused by inhaled industrial silica dust. Whole lung lavage is a useful treatment to remove exogenous dust, inflammatory factors and pro-inflammatory cells from the lungs. The other interstitial lung diseases caused by drugs, autoimmunity, and radiotherapy (rays) are not caused by exogenous dust, so whole lung lavage is rarely used. Meanwhile, the other kinds of BALF samples might not be from large-volume lung lavage (20L). If it is used as a control in this study, its comparability is not strong due to the difference in lung lavage operation. For example, the microbial communities obtained from different oral niches, namely tongue, saliva, and tooth plaque, are different [74, 75]. We tried to use self-control to investigate the effects of “sampling in different rounds of BALF” on its microbiota and mycobiota and to compare the difference between silicosis patients with and without fatigue. Some results may have a close relationship with silicosis patients with fatigue. Future research should further explore more characteristics of the lung microbiome attributable to silicosis.

Second, our sample size was relatively small. This situation is mainly due to three reasons: 1) Subgroups in previous studies contained 3–9 BALF samples [29, 30], indicating that our sample size would be acceptable in this pilot study. 2) In a microbiota study, homogeneity is important to help control confounding factors and improve the precision of the results. We tried our best to select homogeneous patients with the same stage of chronic silicosis. Reasonably, not many homogeneous silicosis patients could meet the inclusion and exclusion requirements. 3) One of our major purposes is to investigate the effects of “sampling in different rounds of BALF” on its microbiota and mycobiota. According to our calculations, the ratio between “microbiota & mycobiota results” and “the number of silicosis” patients should reach 20:1. And we believe approximately 200 “microbiota & mycobiota results” are sufficient in providing appropriate findings and meet the major purpose of this pilot study. Generally, this pilot study with a small sample size is necessary and justifiable, especially in the absence of previous relevant research.

Third, we used only 16S and ITS1 sequencing to analyse the microbiota and mycobiota, respectively. Due to the limitations of PICRUSt2, only functional prediction was performed in our study. Further studies should consider metagenomic detection to validate the functions of BALF microbiota and mycobiota. Fourth, the results may have been affected by factors not assessed in this study, such as the degree of dust concentration exposure and genotypes.

## Conclusion

Silicosis is a debilitating and one of the most fatal work-related diseases. The global prevalence of silicosis warrants further improvement in detection methods. X-rays, spirometry, and high-resolution computed tomography are key procedures for the surveillance of pneumoconiosis and silicosis. However, new techniques, such as microbiome biomarkers, are required. This study used next-generation sequencing technology to sequence and analyse the microbiota and mycobiota in the BALF of silicosis patients, evaluate the impact of “sampling in different rounds of BALF” on its microbiota and mycobiota profiles, and explore the relationship between the microbial and fungal communities and fatigue, a major symptom of silicosis. In conclusion, sampling different rounds of BALF minimally influences the diversity in microbial and fungal communities when the sample amount is sufficient. The first round of BALF collection was recommended for microbial and fungal analyses. BALF microbiota, especially the genus *Vibrio*, could be a potential biomarker and therapeutic target for identifying and alleviating fatigue in patients with silicosis. This research can provide initial insights on microbiome sampling from BALF and future studies focusing on silicosis lung microbiome.

## Supplementary Information

**Additional file 1. **

## Data Availability

All data generated or analysed during this study are included in this published article and its supplementary files. The other datasets used in the current study are available from the corresponding author on reasonable request.

## Abbreviations

BALF: Bronchoalveolar lavage fluid
16 s rRNA: 16S ribosomal ribonucleic acid
ITS1: Internal transcribed spacer 1
FDR: False discovery rate
ASV: Amplicon sequence variants
PCA: Principal component analysis
PCoA: Principal coordinate analysis
LDA: Linear Discriminant Analysis
LefSe: Linear Discriminant Analysis Effect Size Measurement
PICRUSt2: Phylogenetic Investigation of Communities by Reconstruction of Unobserved States 2
ROC: Receiver Operating Characteristic
KEGG: Kyoto Encyclopedia of Genes and Genomes
