# Supplementary material for: Microbiota and mycobiota in bronchoalveolar lavage fluid of silicosis patients
Source: J Occup Med Toxicol. 2023 Jul 10;18:10. doi: 10.1186/s12995-023-00377-3 (PMC10332100; doi:10.1186/s12995-023-00377-3)
Supplement: Supplementary file 1 — Additional file 1. [file 12995_2023_377_MOESM1_ESM.zip › 1_BALF_Supplementary files_Zuo_JOMT_20230622/2-BALF_manuscript_Zuo_JOMT_Supplementary files_20230622.docx]

**Microbiota and Mycobiota in Bronchoalveolar Lavage Fluid of Silicosis Patients**

**Supplementary files**

**Table S1 Participant demographics and clinical characteristics**

| **Items** | **Units** | **no fatigue^1^**  **(n=5)** | **fatigue^2^**  **(n=5)** | **all^3^**  **(n=10)** | ***P* values^4^** |
| --- | --- | --- | --- | --- | --- |
| **Basic Information** |  |  |  |  |  |
| Sex |  |  |  |  | - |
| Male |  | 5 | 5 | 10 |  |
| Female |  | 0 | 0 | 0 |  |
| Age | years | 43.6±6.2 | 46.8±6.0 | 45.2±6.0 | 0.399 |
| Smoking^5^ |  |  |  |  |  |
| Yes |  | 1 | 0 | 1 | - |
| No |  | 4 | 5 | 9 | - |
| Drinking^6^ |  |  |  |  | - |
| Yes |  | 3 | 2 | 5 | - |
| No |  | 2 | 3 | 5 | - |
| **Symptoms** |  |  |  |  |  |
| Cough |  |  |  |  | - |
| Yes |  | 3 | 5 | 8 | - |
| No |  | 2 | 0 | 2 | - |
| Expectoration |  |  |  |  | - |
| Yes |  | 3 | 5 | 8 | - |
| No |  | 2 | 0 | 2 | - |
| Chest pain |  |  |  |  | - |
| Yes |  | 0 | 0 | 0 | - |
| No |  | 5 | 5 | 10 | - |
| Fever |  |  |  |  | - |
| Yes |  | 0 | 0 | 0 | - |
| No |  | 5 | 5 | 10 | - |
| Hemoptysis |  |  |  |  | - |
| Yes |  | 0 | 0 | 0 | - |
| No |  | 5 | 5 | 10 | - |
| **Urinalysis** |  |  |  |  |  |
| Urinary pH^7^ | - | 6.1±0.5 | 6.0±0.8 | 6.1±0.6 | 0.828 |
| Urine specific gravity | - | 1.0±0.0 | 1.0±0.0 | 1.0±0.0 | 0.747 |
| **Routine blood tests** |  |  |  |  |  |
| Red blood cell count | 10^12/L | 5.3±1.1 | 4.8±0.3 | 5.0±0.8 | 0.465 |
| Mean red blood cell volume | fL | 88.0±16.0 | 97.5±2.5 | 92.8±11.9 | 0.173 |
| Mean haemoglobin | pg | 28.8±5.8 | 31.4±0.9 | 30.1±4.1 | 0.465 |
| Mean haemoglobin concentration | g/L | 325.8±9.7 | 322.4±10.3 | 324.1±9.6 | 0.602 |
| Hematocrit determination | % | 45.3±3.8 | 46.6±2.4 | 46.0±3.1 | 0.602 |
| Platelet count | 10^9/L | 175.2±55.0 | 177.2±62.3 | 176.2±55.4 | 0.917 |
| Mean platelet volume | fL | 12.6±2.1 | 12.0±3.1 | 12.3±2.5 | 0.465 |
| White blood cell count | 10^9/L | 5.1±1.1 | 5.3±0.9 | 5.2±1.0 | 0.753 |
| haemoglobin | g/L | 147.6±14.2 | 150.8±12.2 | 149.2±12.6 | 0.834 |
| Red blood cell width - sd value | fL | 41.6±4.5 | 45.5±1.9 | 43.5±3.9 | 0.075 |
| Red blood cell width - cv value | % | 13.2±1.2 | 12.9±0.4 | 13.1±0.9 | 1 |
| Platelet distribution width | - | 16.2±0.4 | 16.2±0.3 | 16.2±0.4 | 0.834 |
| Thrombocytosis | % | 0.2±0.0 | 0.2±0.0 | 0.2±0.0 | 0.292 |
| Neutrophils % | % | 60.6±4.5 | 63.1±8.8 | 61.9±6.7 | 0.173 |
| Lymphocytes % | % | 30.3±3.1 | 27.3±8.2 | 28.8±6.1 | 0.142 |
| Monocytes % | % | 6.1±2.0 | 6.4±1.1 | 6.3±1.5 | 0.347 |
| Eosinophil % | % | 2.5±1.8 | 2.7±1.3 | 2.6±1.5 | 0.602 |
| Basophil % | % | 0.5±0.2 | 0.5±0.2 | 0.5±0.2 | 0.737 |
| Neutrophils# | 10^9/L | 3.1±0.9 | 3.4±0.9 | 3.2±0.8 | 0.465 |
| Lymphocytes# | 10^9/L | 1.5±0.3 | 1.4±0.3 | 1.5±0.3 | 0.602 |
| Monocytes# | 10^9/L | 0.3±0.1 | 0.3±0.1 | 0.3±0.1 | 0.916 |
| Eosinophils# | 10^9/L | 0.1±0.1 | 0.1±0.1 | 0.1±0.1 | 0.344 |
| Prothrombin time | second | 11.3±0.5 | 10.9±0.7 | 11.1±0.6 | 0.346 |
| International normalized ratio | - | 1.0±0.0 | 1.0±0.1 | 1.0±0.1 | 0.344 |
| Partially activated thromboplastin time | second | 34.2±4.7 | 33.7±1.0 | 34.0±3.2 | 0.465 |
| Thrombin time | second | 13.9±0.8 | 14.1±1.1 | 14.0±0.9 | 0.753 |
| Fibrinogen | g/L | 2.5±0.5 | 2.6±0.5 | 2.5±0.5 | 0.834 |
| Total protein | g/L | 71.4±6.0 | 70.3±5.0 | 70.9±5.3 | 0.6 |
| Albumin | g/L | 45.8±3.9 | 43.5±4.7 | 44.6±4.2 | 0.347 |
| Globulin | g/L | 25.7±3.4 | 26.8±2.0 | 26.3±2.7 | 0.754 |
| White ball ratio | - | 1.8±0.2 | 1.6±0.2 | 1.7±0.2 | 0.402 |
| **Blood chemistry tests** |  |  |  |  |  |
| Alanine aminotransferase | U/L | 30.2±23.4 | 29.6±8.6 | 29.9±16.6 | 0.6 |
| Aspartate aminotransferase | U/L | 29.0±13.8 | 30.8±6.2 | 29.9±10.1 | 0.602 |
| Total bilirubin | μmol/L | 12.0±2.0 | 20.0±6.9 | 16.0±6.4 | 0.009 |
| Direct bilirubin | μmol/L | 4.8±0.7 | 7.0±2.3 | 5.9±2.0 | 0.027 |
| Indirect bilirubin | μmol/L | 7.2±1.6 | 13.0±4.6 | 10.1±4.5 | 0.009 |
| Alkaline phosphatase | U/L | 88.0±24.5 | 83.8±29.1 | 85.9±25.4 | 0.6 |
| Gamma-glutamine transpeptidase | U/L | 40.6±26.5 | 42.6±26.3 | 41.6±24.9 | 1 |
| Urea | mmol/L | 6.7±0.6 | 5.9±1.4 | 6.3±1.1 | 0.293 |
| Creatinine | μmol/L | 67.0±10.8 | 63.9±6.1 | 65.5±8.4 | 0.753 |
| Uric acid | μmol/L | 295.7±164.7 | 314.6±50.3 | 305.1±115.3 | 0.465 |
| Blood glucose | mmol/L | 4.9±0.3 | 4.9±0.2 | 4.9±0.2 | 0.917 |
| Cholesterol | mmol/L | 4.6±0.4 | 5.2±0.7 | 4.9±0.6 | 0.142 |
| Triglycerides | mmol/L | 1.1±0.5 | 1.3±0.6 | 1.2±0.5 | 0.599 |
| High density lipoprotein | mmol/L | 1.5±0.2 | 1.3±0.2 | 1.4±0.2 | 0.295 |
| Low density lipoprotein | mmol/L | 2.8±0.4 | 3.6±0.8 | 3.2±0.7 | 0.117 |
| **Hepatitis B serologic testing** |  |  |  |  |  |
| Hepatitis B surface antigen | IU/mL | 125.9±278.3 | 0.0±0.0 | 62.9±197.0 | 0.136 |
| Hepatitis B surface antibody | mIU/mL | 156.4±239.5 | 140.3±223.5 | 148.4±218.6 | 0.751 |
| Hepatitis B e antigen | pEIU/mL | 8.5±18.9 | 0.0±0.0 | 4.2±13.4 | 0.317 |
| Hepatitis B e antibody | pEIU/mL | 1.9±2.3 | 0.2±0.2 | 1.0±1.8 | 0.251 |
| Hepatitis B core antibody | IU/mL | 4.5±2.3 | 2.2±1.7 | 3.3±2.2 | 0.117 |
| **Silicosis status / radiological classification** |  |  |  |  |  |
| Chronic simple silicosis | - | 5 | 5 | 10 | - |
| Chronic complicated silicosis | - | 0 | 0 | 0 | - |
| Accelerated silicosis | - | 0 | 0 | 0 | - |
| Acute silicosis | - | 0 | 0 | 0 | - |
| **Lung function** |  |  |  |  |  |
| FVC%Pred^8^ | % | 91.9±28.0 | 85.5±17.3 | 88.7±22.2 | 0.675 |
| FEV1%Pred^9^ | % | 91.0±22.3 | 90.9±8.2 | 90.9±15.9 | 0.994 |
| FEV1%FVC Pred^10^ | % | 99.3±6.8 | 106.3±12.7 | 102.8±10.3 | 0.310 |
| DLCO%Pred^11^ | % | 80.8±21.5 | 84.5±24.2 | 82.6±21.7 | 0.806 |
| **Hemogasanalysis** |  |  |  |  |  |
| pH^12^ | - | 7.4±0.0 | 7.4±0.0 | 7.4±0.0 | 0.466 |
| sO_2_^13^ | % | 96.4±1.1 | 95.6±0.9 | 96.0±1.1 | 0.252 |
| pO_2_^14^ | mmHg | 99.6±5.6 | 98.0±4.9 | 98.8±5.1 | 0.645 |
| pCO_2_^15^ | mmHg | 40.8±3.4 | 37.4±2.1 | 39.1±3.2 | 0.094 |

1, no fatigue: silicosis patients without fatigue; 2, fatigue: silicosis patients with fatigue; 3, all: all ten patients; 4, rank sum test, when *P* value < 0.05, the values are in red; pEIU: Paul Ehrlich international units; mIU: milli-International Units; /HP: per high power field; 5, Smoking, Yes: current smoker + ever smoker; 6, Drinking, Yes: current drinking + ever drinking; 7, Urinalysis, urine dry chemistry test; 8, FVC, Forced vital capacity; 9, Pred, predicted; 10, FEV1, forced expiratory volume in 1 second; 11, DLCO, diffusing capacity of the lung for carbon monoxide; 12, pH, acidity; 13, sO_2_, Oxygen saturation; 14, pO_2_, partial pressure of oxygen; 15, pCO_2_, partial pressure of carbon dioxide.

**Table S2 KEGG pathway analysis using PICRUST2 among silicosis patients (level1, level2, level3).**

Data is available at <https://pan.baidu.com/s/1tkXeuWJBroKBdS3ormqH0Q> (Password: 566i).

**Table S3 PICRUST2 microbial enzyme prediction among silicosis patients (top 50 of the most abundant enzymes).**

Data is available at <https://pan.baidu.com/s/1tkXeuWJBroKBdS3ormqH0Q> (Password: 566i).

**Table S4 PICRUST2 fungal enzyme prediction among silicosis patients (top 50 of the most abundant enzymes).**

The images of the Supplementary files are large, please see the download link below:

Data is available at <https://pan.baidu.com/s/1tkXeuWJBroKBdS3ormqH0Q> (Password: 566i).


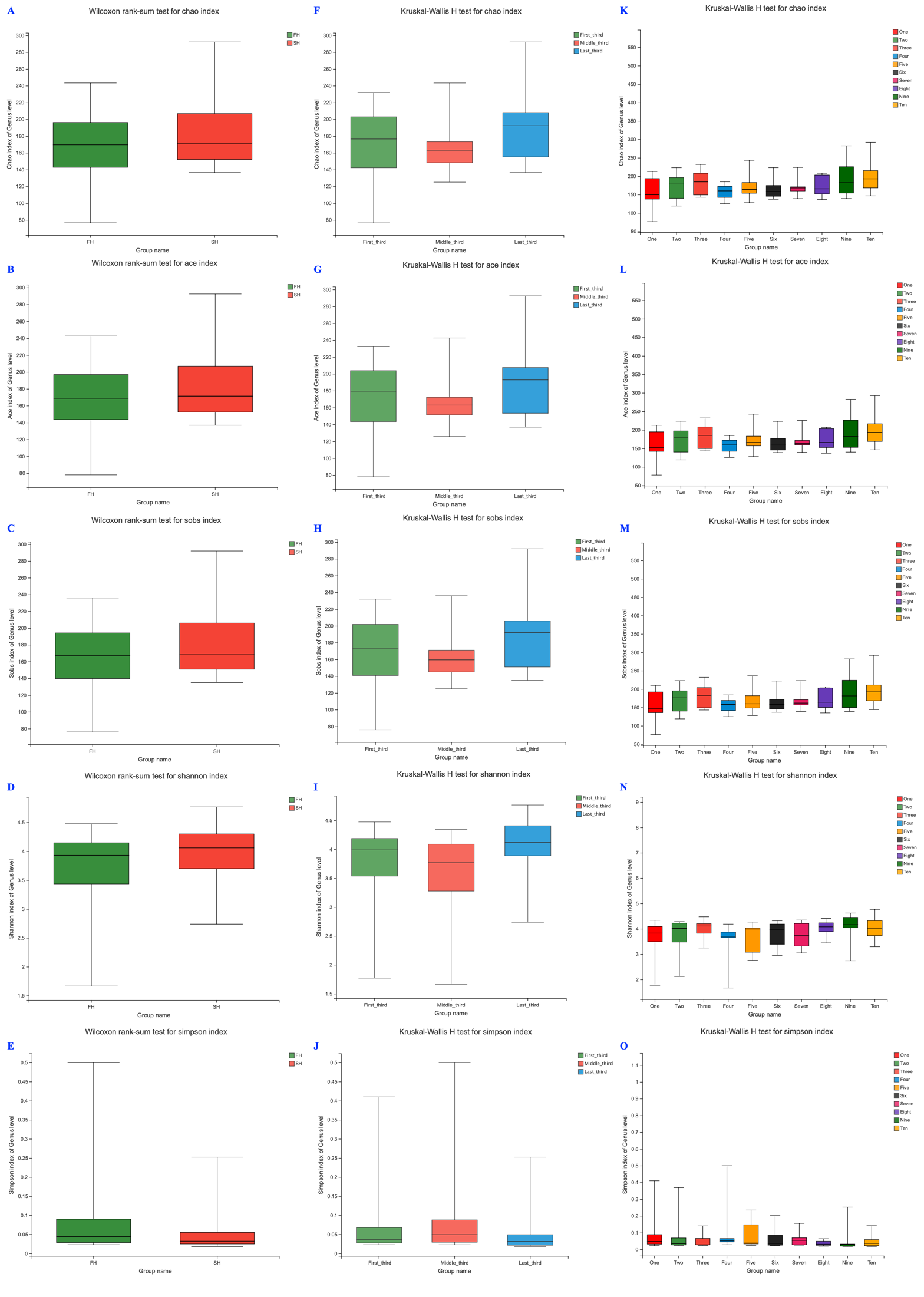


**Figure S1 BALF microbiota α diversity of silicosis patients**. The BALF samples from rounds 1 to 10 were divided into 2 groups, namely: the first five rounds (first half, FH, 50%) and the last five rounds (second half, SH, 50%). A-E: α diversity analyses between the FH group and the SH group (n=81). The BALF samples from rounds 1 to 10 were divided into 3 groups, namely: the fisrt third (30%), the middle third (40%) and the last third (30%). F-J: α diversity analyses among the fisrt third, the middle third and the last third groups (n=81). K-O: α diversity analyses of BALF samples from rounds 1-10 were conducted (n=81).

**
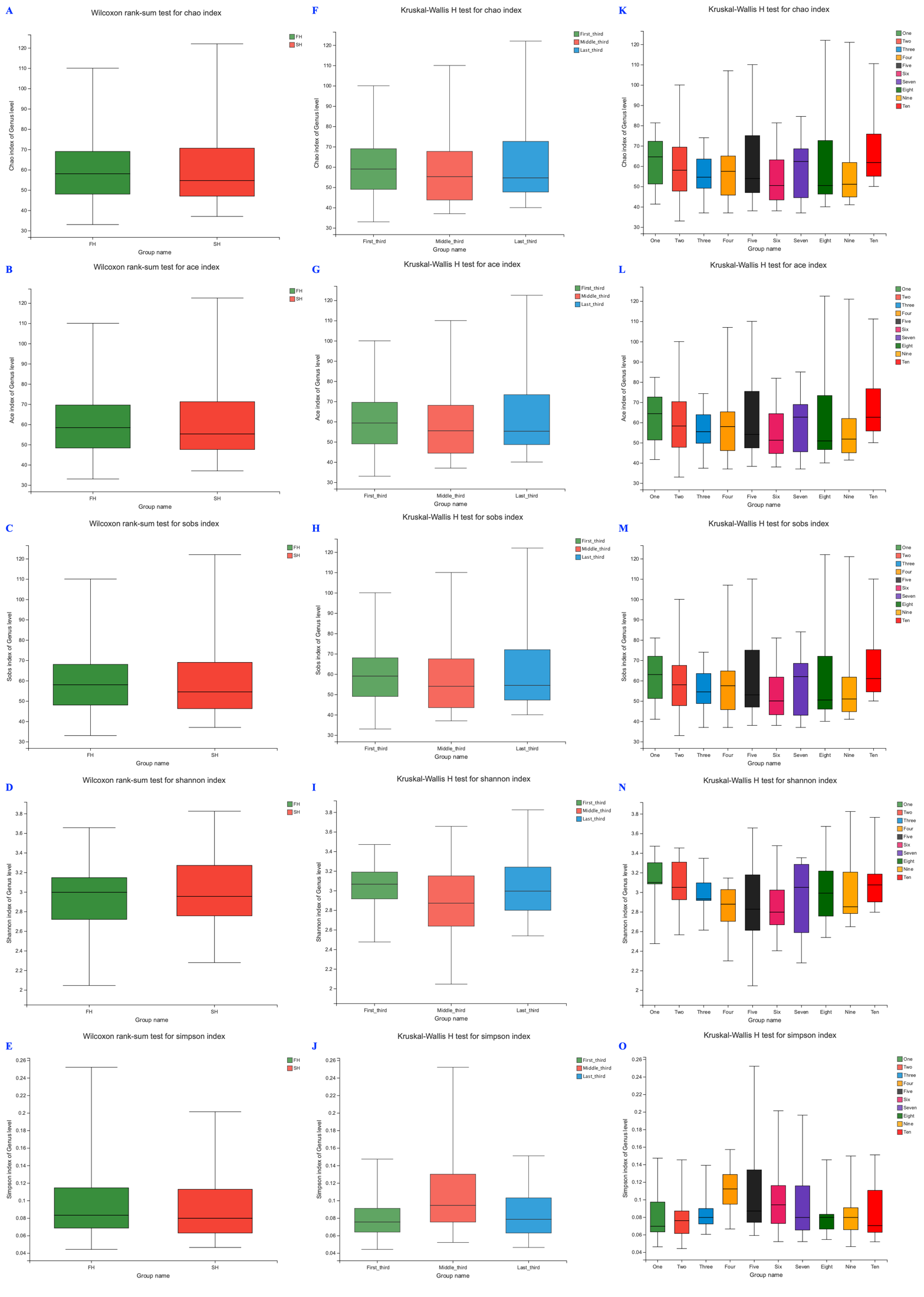
**

**Figure S2 BALF mycobiota α diversity of silicosis patients**. The BALF samples from rounds 1 to 10 were divided into 2 groups, namely: the first five rounds (first half, FH, 50%) and the last five rounds (second half, SH, 50%). A-E: α diversity analyses between the FH group and the SH group (n=100). The BALF samples from rounds 1 to 10 were divided into 3 groups, namely: the first third (30%), the middle third (40%) and the last third (30%). F-J: α diversity analyses among the first third, the middle third and the last third groups (n=100). K-O: α diversity analyses of BALF samples from rounds 1-10 were conducted (n=100).


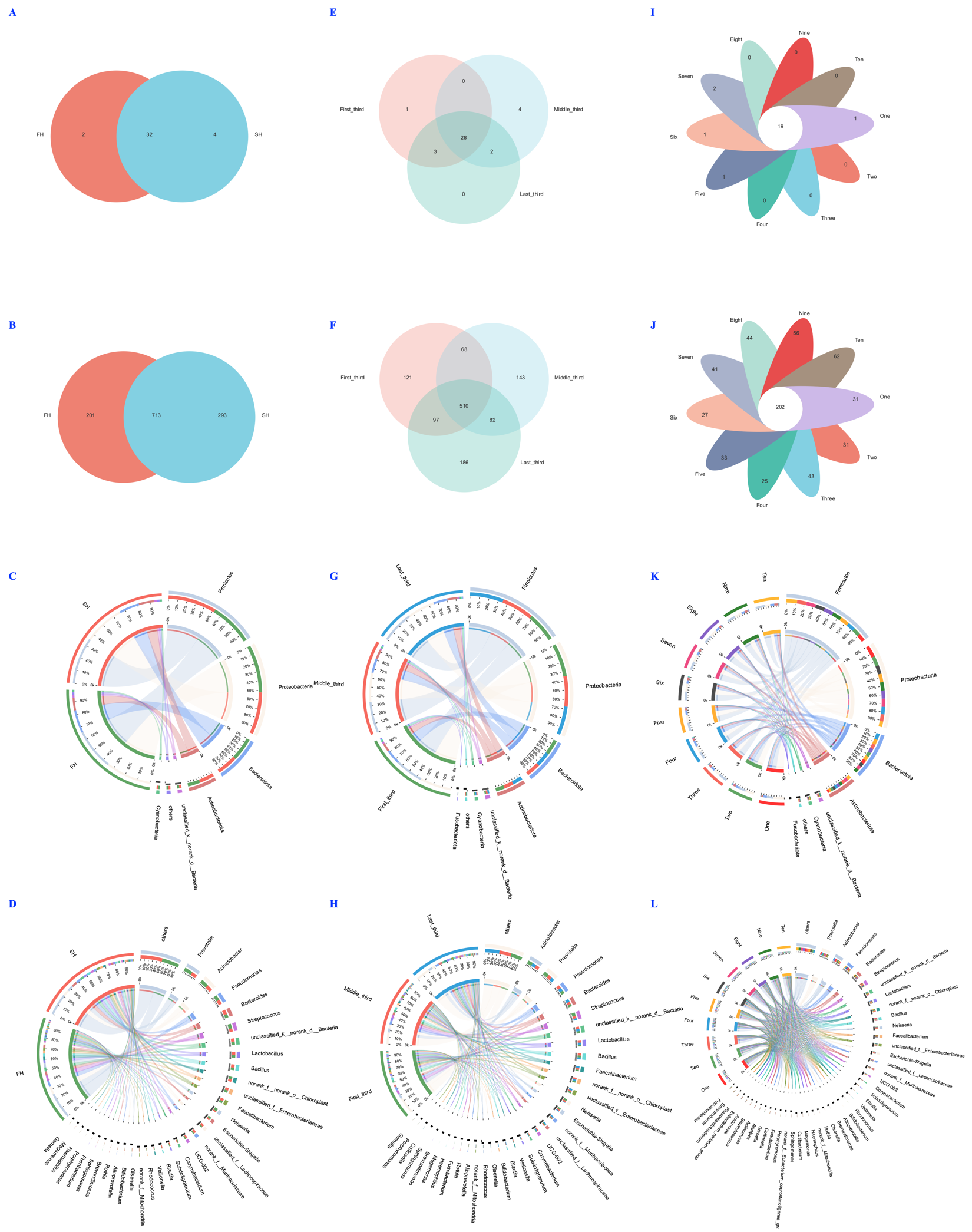


**Figure S3 Venn and circus analyses of BALF microbiota in silicosis patients**. Firstly, the BALF samples from rounds 1 to 10 were divided into 2 groups, namely: the first five rounds (first half, FH, 50%) and the last five rounds (second half, SH, 50%, Figure S3A-S3D). Secondly, the BALF samples from rounds 1 to 10 were divided into 3 groups, namely: the first third (30%), the middle third (40%) and the last third (30%, Figure S3E-S3H). Thirdly, the BALF samples from rounds 1 to 10 were divided into 10 groups (Figure S3I-S3L). A, B, E, F, I, J: Venn analyses (n=81); C, D, G, H, K, L: circus analyses (n=81).


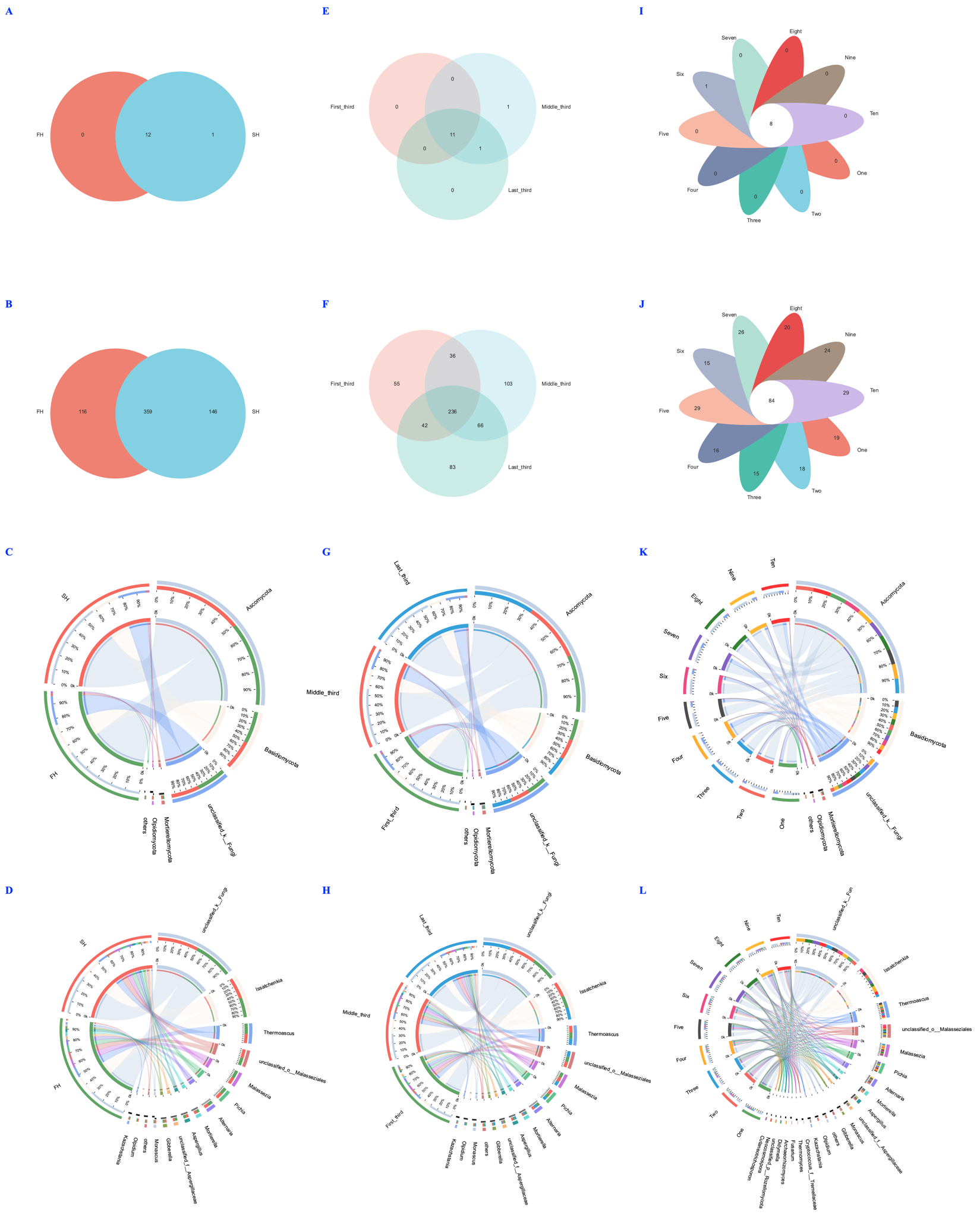


**Figure S4 Venn and circus analyses of BALF mycobiota in silicosis patients**. Firstly, the BALF samples from rounds 1 to 10 were divided into 2 groups, namely: the first five rounds (first half, FH, 50%) and the last five rounds (second half, SH, 50%, Figure S4A-S4D). Secondly, the BALF samples from rounds 1 to 10 were divided into 3 groups, namely: the first third (30%), the middle third (40%) and the last third (30%, Figure S4E-S4H). Thirdly, the BALF samples from rounds 1 to 10 were divided into 10 groups (Figure S4I-S4L). A, B, E, F, I, J: Venn analyses (n=100); C, D, G, H, K, L: circus analyses (n=100).


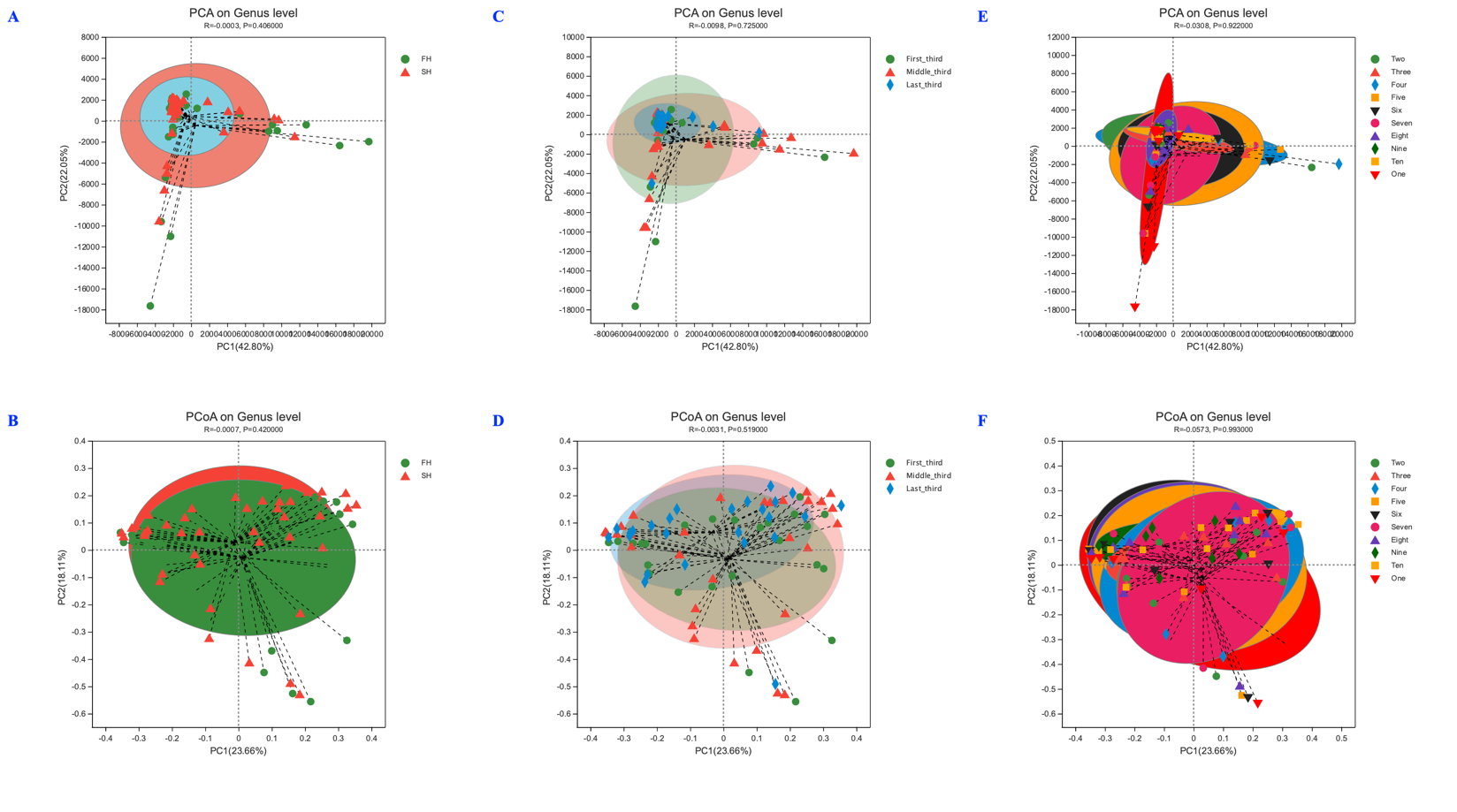


**Figure S5 PCA and PCoA analyses of BALF microbiota in silicosis patients**. Firstly, the BALF samples from rounds 1 to 10 were divided into 2 groups, namely: the first five rounds (first half, FH, 50%) and the last five rounds (second half, SH, 50%, Figure S5A-S5B). Secondly, the BALF samples from rounds 1 to 10 were divided into 3 groups, namely: the first third (30%), the middle third (40%) and the last third (30%, Figure S5C-S5D). Thirdly, the BALF samples from rounds 1 to 10 were divided into 10 groups (Figure S5E-S5F). A, C, E: PCA analyses (n=81); B, D, F: PCoA analyses (n=81).


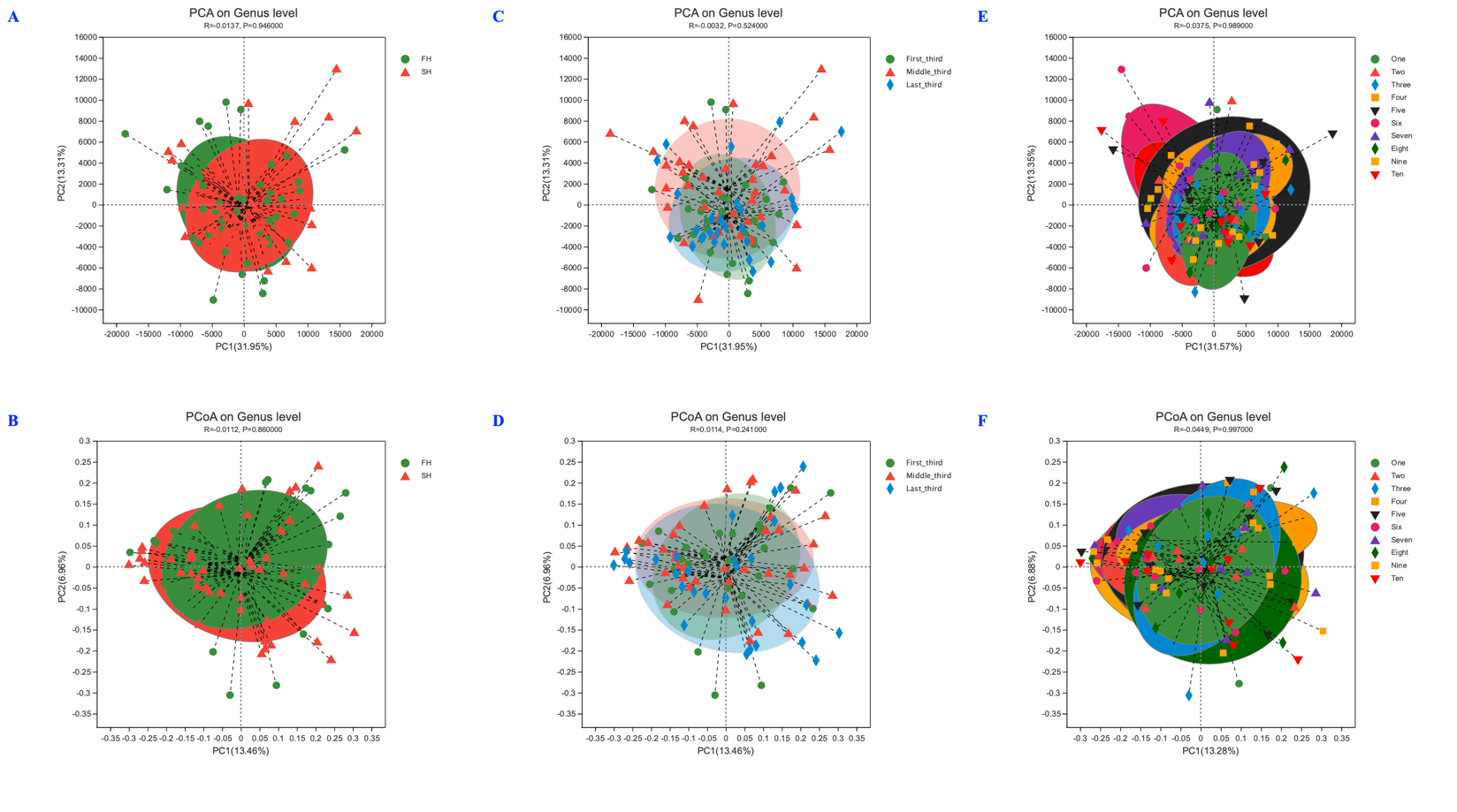


**Figure S6 PCA and PCoA analyses of BALF mycobiota in silicosis patients**. Firstly, the BALF samples from rounds 1 to 10 were divided into 2 groups, namely: the first five rounds (first half, FH, 50%) and the last five rounds (second half, SH, 50%, Figure S6A-S6B). Secondly, the BALF samples from rounds 1 to 10 were divided into 3 groups, namely: the first third (30%), the middle third (40%) and the last third (30%, Figure S6C-S6D). Thirdly, the BALF samples from rounds 1 to 10 were divided into 10 groups (Figure S6E-S6F). A, C, E: PCA analyses (n=100); B, D, F: PCoA analyses (n=100).


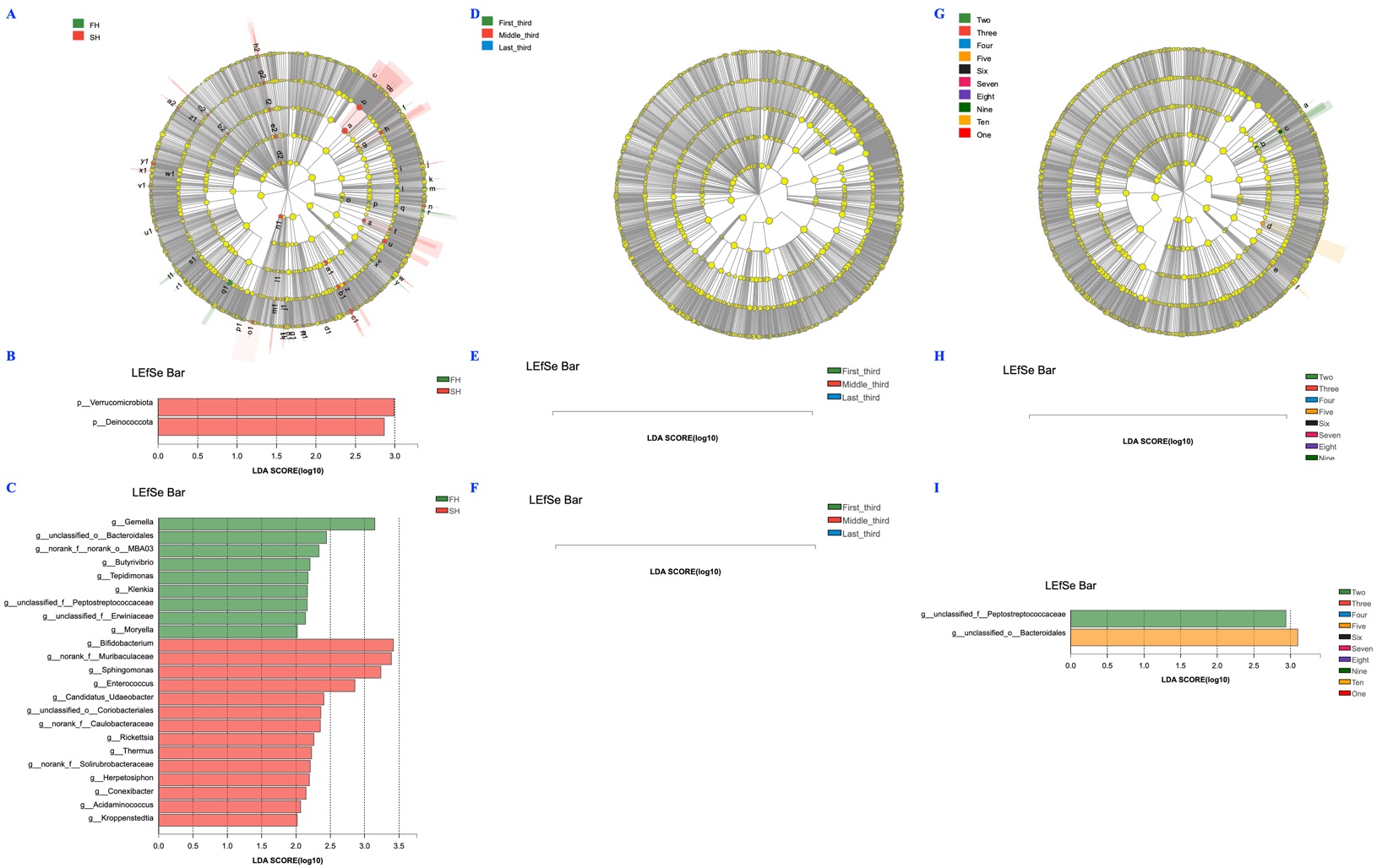


**Figure S7 LEfSe analyses of BALF microbiota in silicosis patients**. Firstly, the BALF samples from rounds 1 to 10 were divided into 2 groups, namely: the first five rounds (first half, FH, 50%) and the last five rounds (second half, SH, 50%, Figure S7A-S7B). Secondly, the BALF samples from rounds 1 to 10 were divided into 3 groups, namely: the first third (30%), the middle third (40%) and the last third (30%, Figure S7C-S7D). Thirdly, the BALF samples from rounds 1 to 10 were divided into 10 groups (Figure S7E-S7F). A, D, G: Cladograms of LefSe (n=81); B, E, H: LefSe analysis at the phylum level (n=81); C, F, I: LefSe analysis at the genus level (n=81).


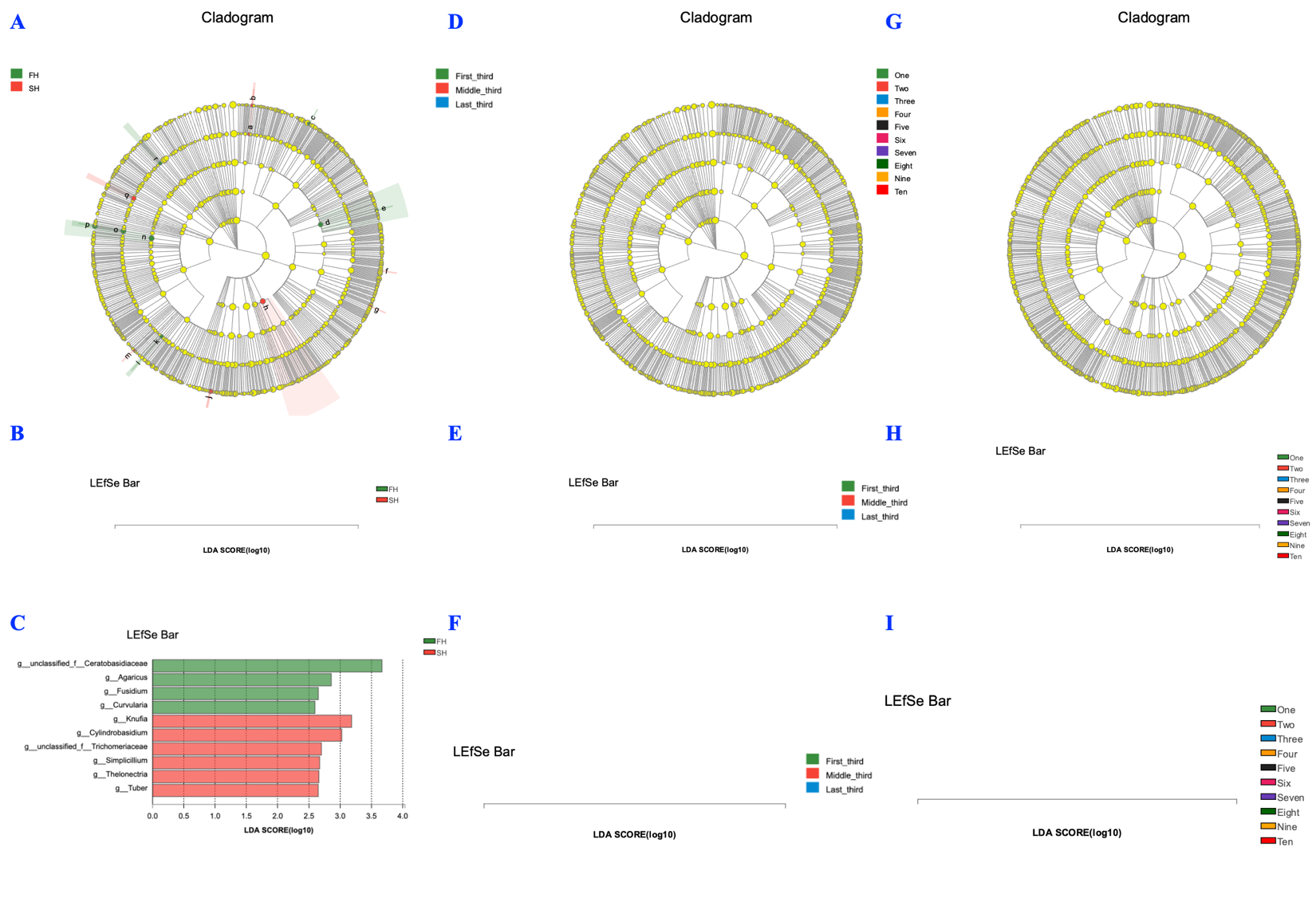


**Figure S8 LEfSe analyses of BALF mycobiota in silicosis patients**. Firstly, the BALF samples from rounds 1 to 10 were divided into 2 groups, namely: the first five rounds (first half, FH, 50%) and the last five rounds (second half, SH, 50%, Figure S8A-S8B). Secondly, the BALF samples from rounds 1 to 10 were divided into 3 groups, namely: the first third (30%), the middle third (40%) and the last third (30%, Figure S8C-S8D). Thirdly, the BALF samples from rounds 1 to 10 were divided into 10 groups (Figure S8E-S8F). A, D, G: Cladograms of LefSe (n=100); B, E, H: LefSe analysis at the phylum level (n=100); C, F, I: LefSe analysis at the genus level (n=100).


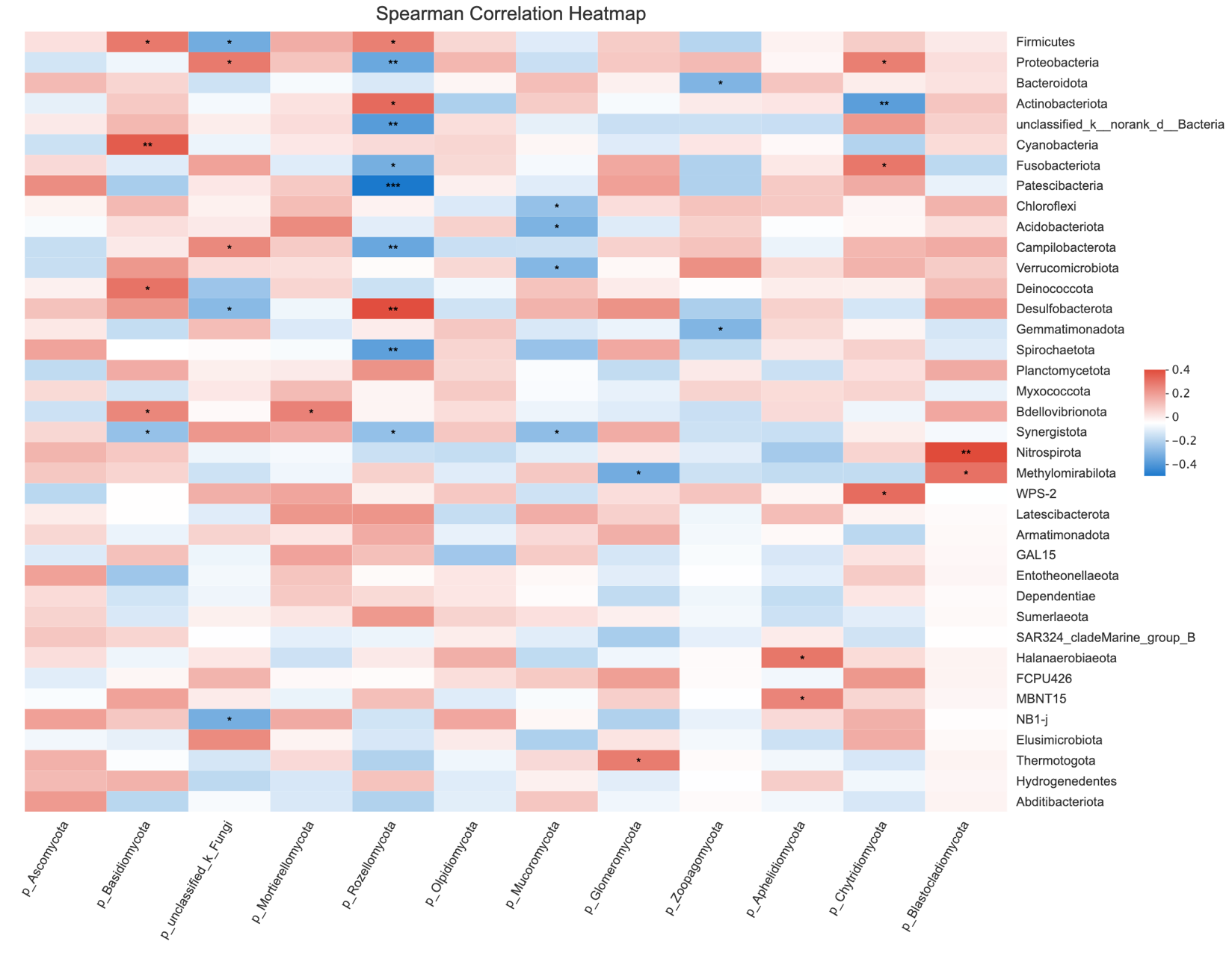


**Figure S9 Microbiota_mycobiota interaction at the phylum level**.


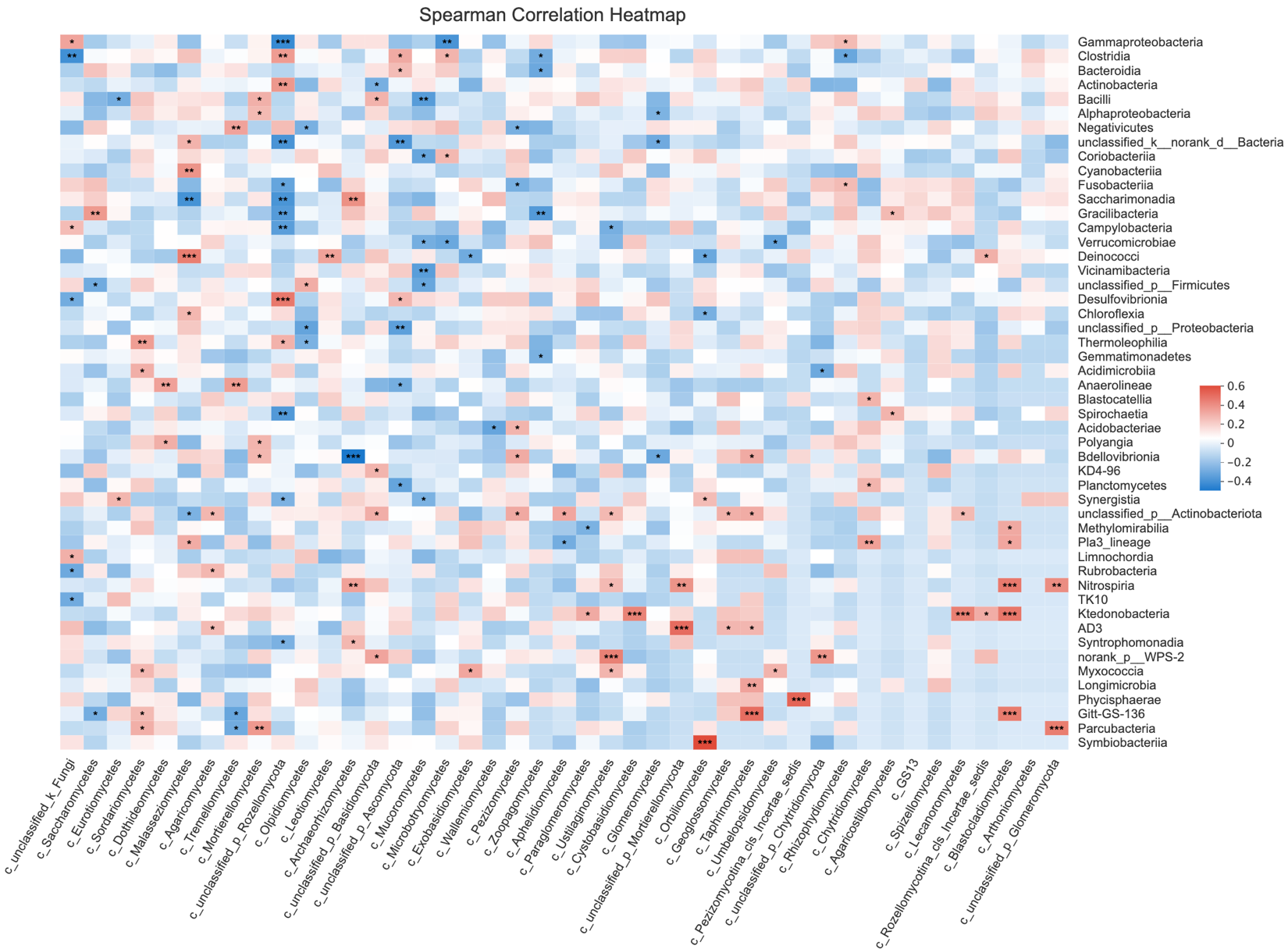


**Figure S10 Microbiota_mycobiota interaction at the class level**.


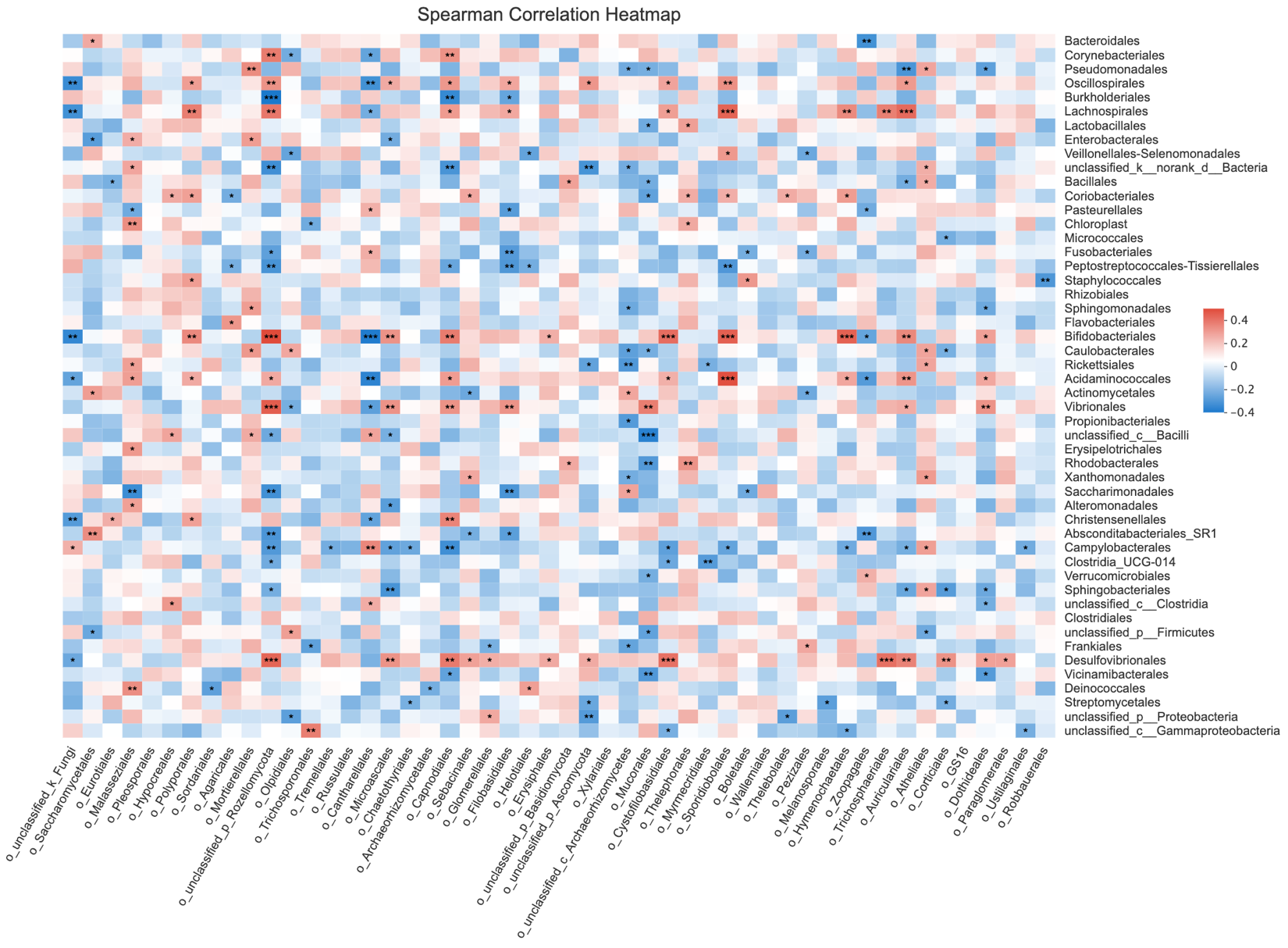


**Figure S11 Microbiota_mycobiota interaction at the order level**.


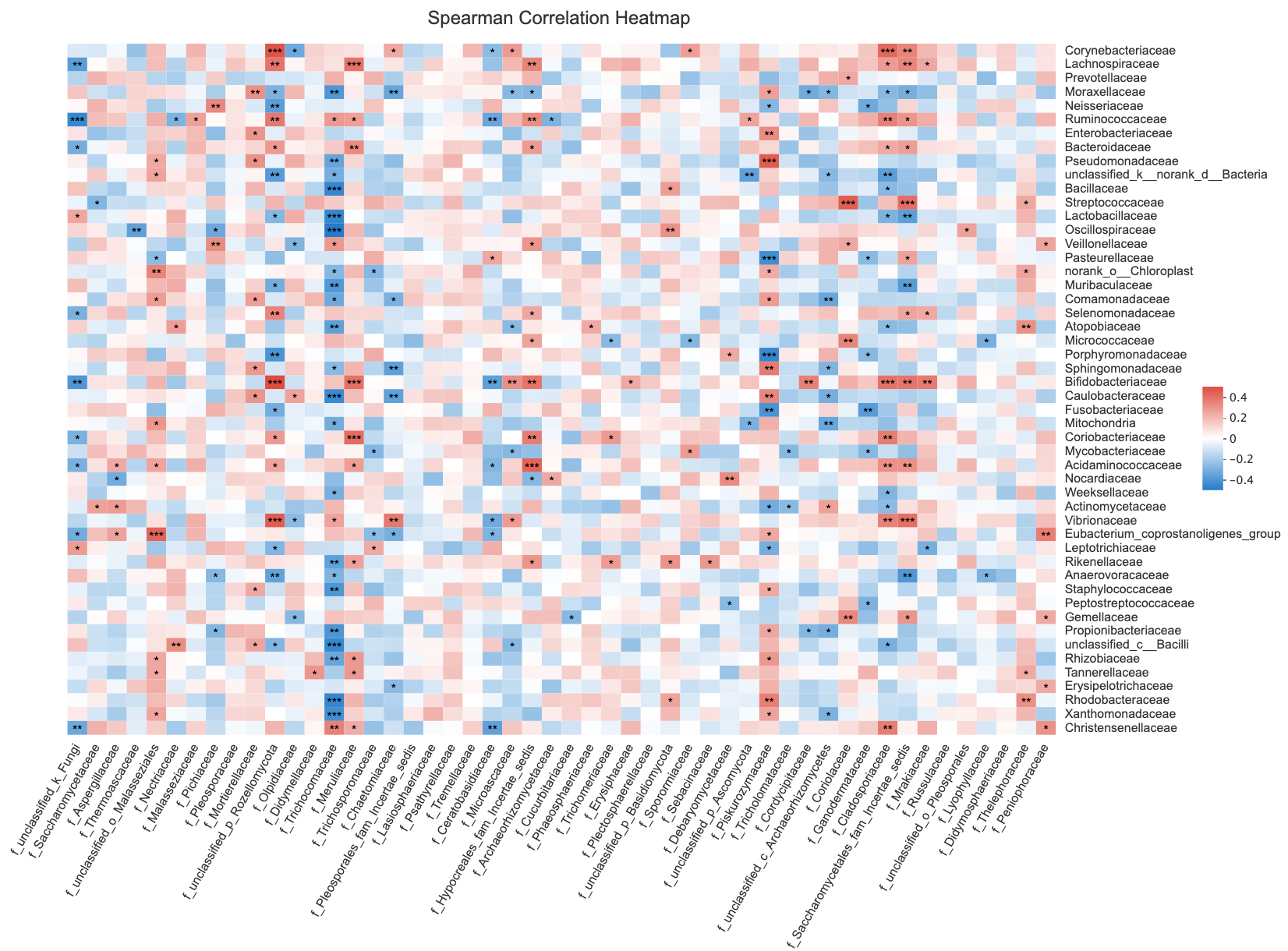


**Figure S12 Microbiota_mycobiota interaction at the family level.**


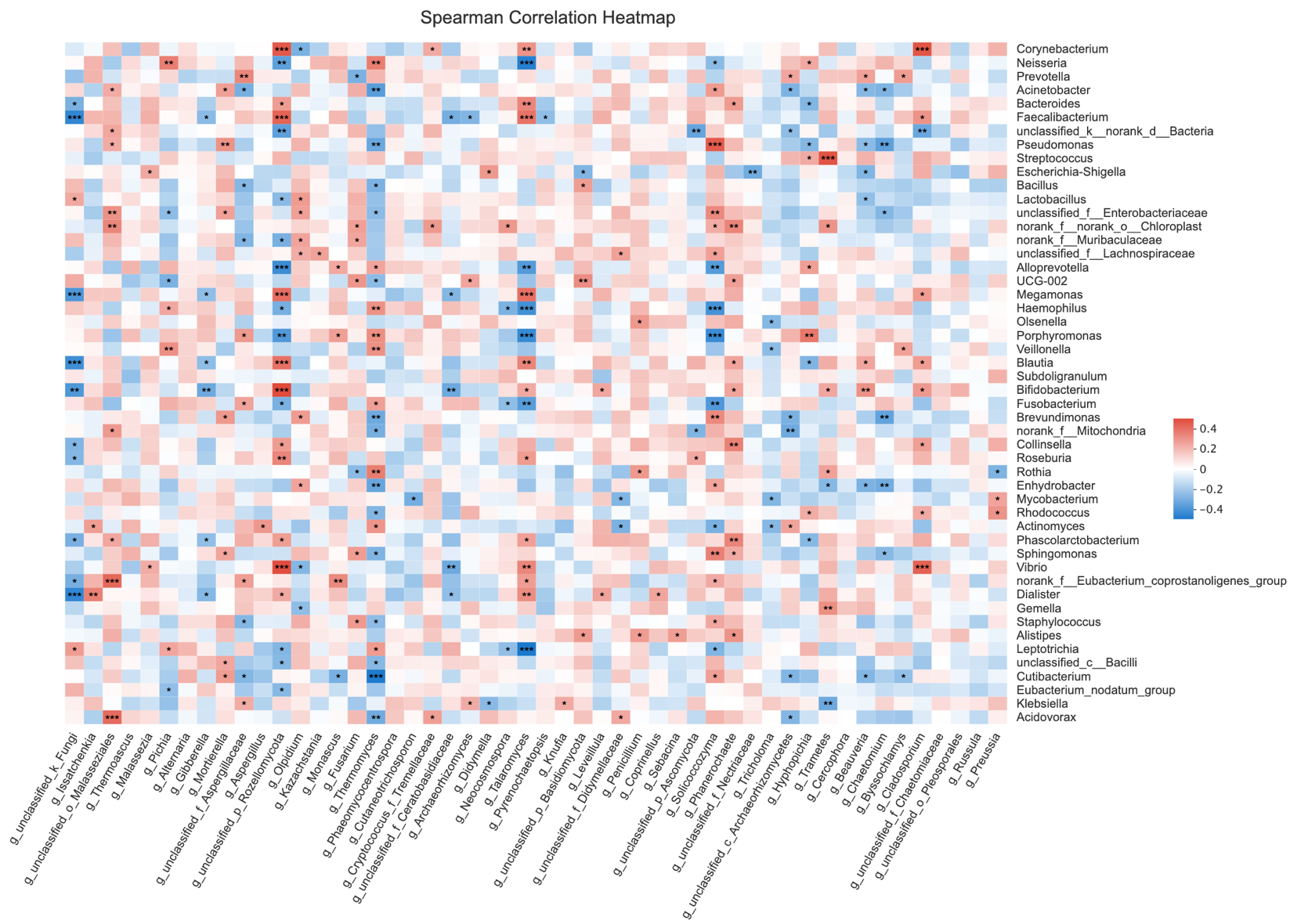


**Figure S13** **Microbiota_mycobiota interaction at the genus level.**

**
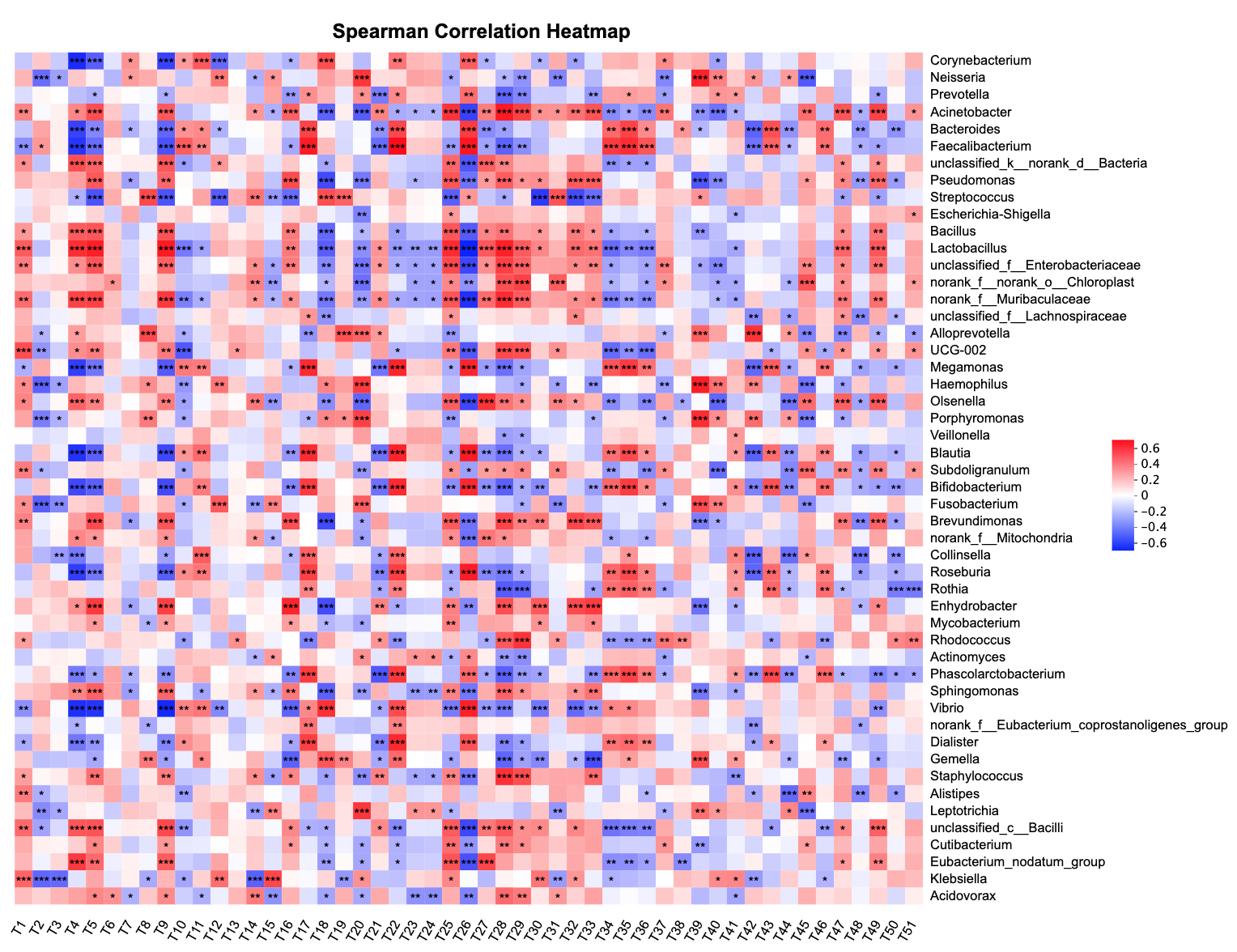
**

**Figure S14** **Microbiota_Blood test interaction at the genus level.**

T1_Red blood cell count, T2_Mean red blood cell volume, T3_Mean hemoglobin, T4_Mean hemoglobin concentration, T5_Hematocrit determination, T6_Platelet count, T7_Mean platelet volume, T8_White blood cell count, T9_Hemoglobin, T10_Red blood cell width - sd value, T11_Red blood cell width - cv value, T12_Platelet distribution width, T13_Thrombocytosis, T14_Neutrophils %, T15_Lymphocytes %, T16_Monocytes %, T17_Eosinophil %, T18_Basophil %, T19_Neutrophils#, T20_Lymphocytes#, T21_Monocytes#, T22_Eosinophils#, T23_Prothrombin time, T24_International normalized ratio, T25_Partially activated thromboplastin time, T26_Thrombin time, T27_Fibrinogen, T28_Total protein, T29_Albumin, T30_Globulin, T31_White ball ratio, T32_Alanine aminotransferase, T33_Aspartate aminotransferase, T34_Total bilirubin, T35_Direct bilirubin, T36_Indirect bilirubin, T37_Alkaline phosphatase, T38_Gamma-glutamine transpeptidase, T39_Urea, T40_Creatinine, T41_Uric acid, T42_Blood glucose, T43_Cholesterol, T44_Triglycerides, T45_High density lipoprotein, T46_Low density lipoprotein, T47_Hbsag, T48_Hepatitis b surface antibody, T49_Hepatitis b e antigen, T50_Hepatitis b e antibody, T51_Hepatitis b core antibody.

**
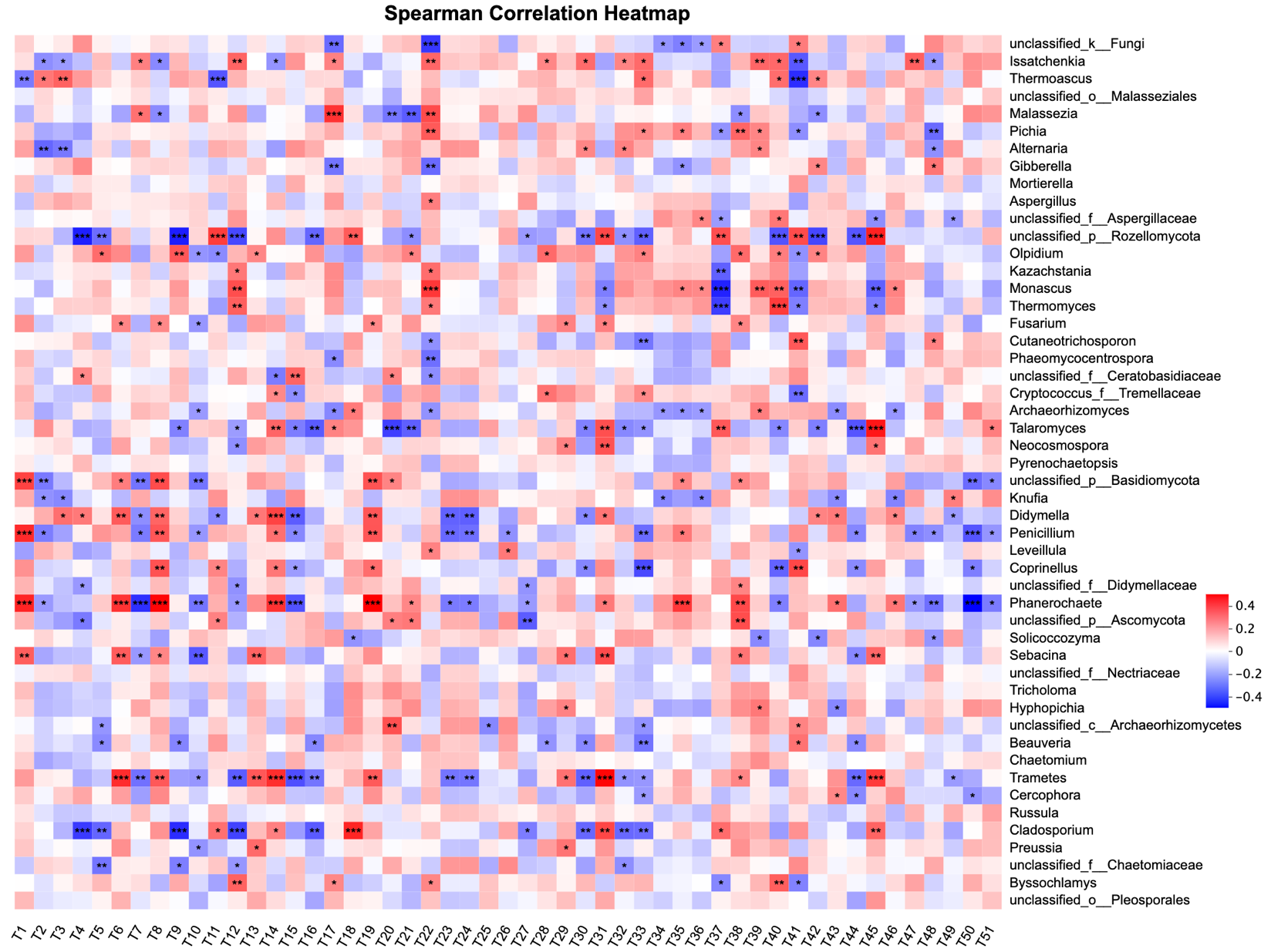
**

**Figure S15** **Mycobiota_Blood test interaction at the genus level.**

T1_Red blood cell count, T2_Mean red blood cell volume, T3_Mean hemoglobin, T4_Mean hemoglobin concentration, T5_Hematocrit determination, T6_Platelet count, T7_Mean platelet volume, T8_White blood cell count, T9_Hemoglobin, T10_Red blood cell width - sd value, T11_Red blood cell width - cv value, T12_Platelet distribution width, T13_Thrombocytosis, T14_Neutrophils %, T15_Lymphocytes %, T16_Monocytes %, T17_Eosinophil %, T18_Basophil %, T19_Neutrophils#, T20_Lymphocytes#, T21_Monocytes#, T22_Eosinophils#, T23_Prothrombin time, T24_International normalized ratio, T25_Partially activated thromboplastin time, T26_Thrombin time, T27_Fibrinogen, T28_Total protein, T29_Albumin, T30_Globulin, T31_White ball ratio, T32_Alanine aminotransferase, T33_Aspartate aminotransferase, T34_Total bilirubin, T35_Direct bilirubin, T36_Indirect bilirubin, T37_Alkaline phosphatase, T38_Gamma-glutamine transpeptidase, T39_Urea, T40_Creatinine, T41_Uric acid, T42_Blood glucose, T43_Cholesterol, T44_Triglycerides, T45_High density lipoprotein, T46_Low density lipoprotein, T47_Hbsag, T48_Hepatitis b surface antibody, T49_Hepatitis b e antigen, T50_Hepatitis b e antibody, T51_Hepatitis b core antibody.
